# Childhood adversity and disadvantage in relation to incident disability and multimorbidity: prospective analyses of ELSA and two SHARE inception samples

**DOI:** 10.3389/fpubh.2026.1889565

**Published:** 2026-09-08

**Authors:** Tao Zhou, Guohua Jiang, Ruijinlin Hao, Yi Fang, Li Liu, Jun Wu, Xiaojin Zhang

**Affiliations:** 1 Department of Geriatrics, The First Affiliated Hospital with Nanjing Medical University, Nanjing, China; 2 Department of Anesthesiology, The First Affiliated Hospital with Nanjing Medical University, Nanjing, China; 3 Department of Geriatric Cardiology, Jiangsu Provincial Key Laboratory of Geriatrics, The First Affiliated Hospital with Nanjing Medical University, Nanjing, China

**Keywords:** activities of daily living, childhood adversity, childhood socioeconomic disadvantage, disability, ELSA, life-course epidemiology, multimorbidity, SHARE

## Abstract

**Background:**

Childhood health adversity and socioeconomic disadvantage may shape late-life health, but evidence for incident disability and multimorbidity remains limited. Cross-cohort inference is complicated by non-equivalent measures of childhood experiences and differences in timing. We estimated cohort-specific prospective associations in the English Longitudinal Study of Ageing (ELSA) and the Survey of Health, Ageing and Retirement in Europe (SHARE).

**Methods:**

ELSA childhood-health/material-adversity indicators and health were assessed at wave 3. SHARE material/cultural disadvantage was assessed at wave 3 or wave 7, with health landmarks at waves 4 and 8; the two inception samples were analyzed separately. Outcomes were the first observed onset of difficulty in at least one of six activities of daily living (ADL-6) and the first observed onset of at least two of seven disease groups (MM-7). Complementary log–log person–period models incorporated interval duration, repeated observations, available survey-design information and baseline weights, and stabilized inverse-probability-of-censoring weights; death was a competing event. Highest exposure categories were compared with zero, without assuming measurement equivalence.

**Results:**

Combined survey/attrition-weighted models included 4,290/3,424 ELSA participants, 13,863/9,064 SHARE wave-3 participants, and 23,026/12,560 SHARE wave-7 participants for ADL-6/MM-7, respectively. In the mortality-ascertained ELSA wave 4–6 window, highest-versus-zero hazard ratios (HRs) were 1.43 (95% confidence interval [CI] 1.09–1.87) for ADL-6 and 1.35 (1.02–1.80) for MM-7. After excluding ADL-6 onset observed at wave 4, the lagged ELSA HR was 1.03 (0.70–1.52) through wave 6. Over waves 4–9, HRs were 1.47 (1.19–1.82) and 1.24 (0.98–1.57). SHARE wave-3 HRs were 1.36 (1.08–1.71) and 1.34 (1.09–1.64); wave-7 HRs were 1.60 (1.06–2.43) and 0.93 (0.68–1.27). Standardized competing-risk estimates showed a higher risk of ADL-6 in the primary ELSA analysis and both SHARE inception-sample analyses. MM-7 risk differences were positive in ELSA and SHARE wave 3, whereas the SHARE wave-7 contrast was imprecise and compatible with no association.

**Conclusion:**

The highest cohort-specific exposure category versus zero was associated with incident ADL disability in the primary ELSA and SHARE landmark analyses, but the ELSA association appeared strongest in the earliest interval and was not clearly sustained later. Associations with multimorbidity varied by observation window and SHARE inception sample. The findings neither establish measurement equivalence nor explain the observed cross-cohort heterogeneity.

## Introduction

Adverse childhood experiences and socioeconomic disadvantage have been associated with poorer physical health, chronic disease, and mortality across adulthood (1–3). Life-course epidemiology proposes that early exposures may influence later health through biological embedding, developmental disruption, and the accumulation or continuity of social disadvantage (4, 5). A recent systematic review and a 70-year national cohort study linked adverse childhood experiences with multimorbidity or its accumulation across adulthood (6, 7); studies of childhood socioeconomic conditions likewise suggest associations with multimorbidity in later life, although the evidence is heterogeneous and methodologically limited (8, 9). Prospective evidence also links cumulative childhood adversity with less favorable trajectories of activities of daily living (ADL) disability (10). These pathways and findings are relevant to ADL disability and multimorbidity, two common and consequential features of population aging (11, 12).

Important uncertainties remain. Much of the adversity literature focuses on single countries, cross-sectional late-life outcomes, or broad adult-health composites. Prospective evidence on the first observed onset of late-life ADL disability and multimorbidity remains comparatively limited and methodologically heterogeneous (7–10). Analyses based only on status at the final available follow-up can obscure when an outcome arose, ignore intermittent non-response and unequal observation periods, and treat deaths before outcome ascertainment as if they were ordinary missing observations. These issues are material in aging cohorts, in which attrition and mortality are strongly structured by age and health.

Cross-cohort comparison introduces a separate conceptual problem. Adverse events, childhood health, material deprivation, cultural resources, and parental education are related but non-interchangeable indicators. Differences in their prevalence or estimated associations cannot automatically be interpreted as differences in a common exposure effect. Cross-national comparisons of later-life health also reflect differences in population composition, institutional context, measurement, and harmonization (13–15). Retrospective childhood reports create further scope for discordance, recall error, cohort effects, and selection (16, 17). Consequently, a defensible multi-cohort analysis must preserve cohort-specific meanings and timing rather than impose a common score scale.

The present study focuses on the prospective evidence available in ELSA and SHARE. Analyses based on non-equivalent exposure proxies or insufficiently aligned longitudinal landmarks were outside its evidential scope. The contribution is substantive and design-oriented: to estimate cohort-specific associations between early-life conditions and first observed late-life ADL disability and multimorbidity while explicitly accounting for exposure timing, repeated observations, unequal follow-up, attrition, available survey-design information, and competing mortality.

We asked whether cohort-specific measures of childhood adversity in ELSA and childhood material/cultural disadvantage in two separately timed SHARE inception samples were associated with the first observed onset of ADL-6 disability and MM-7 multimorbidity. We also estimated standardized absolute risks in the presence of competing mortality. We did not assume measurement invariance across cohorts, directly compare the magnitude of the ELSA and SHARE coefficients as a common dose–response effect, or attempt to identify the mechanisms underlying between-sample differences.

## Materials and methods

### Study design and analysis plan

We conducted prospective secondary analyses of ELSA and SHARE. ELSA is a longitudinal study of community-dwelling adults aged 50 years or older in England, initially sampled from households that had participated in the Health Survey for England, with approximately biennial interviews (18). SHARE uses country-specific probability samples and follows adults aged 50 years or older and their partners in multiple European countries and Israel; sampling frames and fieldwork designs vary by country (19).

The final analysis plan was developed after peer review, dated 23 July 2026, and accompanied by a dated amendment record; it was not prospectively registered or pre-specified.

### Cohorts, landmarks, and follow-up

#### ELSA

The ELSA wave-3 life-history interview (2006–2007) was the childhood-exposure assessment and health landmark. Eligible participants completed the life-history module, responded to the wave-3 main interview, were aged at least 50 years, had all three childhood indicators observed, and were free of the outcome under analysis at wave 3. Follow-up outcome assessments were conducted at waves 4–9 (2008–2019).

Locally available mortality data were complete through wave 6. We therefore treated waves 4–6, representing up to approximately 6 years, as the mortality-ascertained ELSA window. Waves 4–9, representing up to approximately 12 years of outcome-status follow-up, were analyzed as an extended sensitivity window with explicitly incomplete mortality ascertainment after wave 6.

#### SHARE wave-3 inception sample

For participants first assessed in the wave-3 SHARELIFE interview (2008–2009), childhood exposure was measured at wave 3. The SHARELIFE interview did not provide a same-wave regular health assessment comparable with the later core interviews; wave 4 (2010–2011) was therefore the health landmark and waves 5–9 were follow-up. The exposure-complete landmark sample comprised participants aged at least 50 years from 12 countries.

#### SHARE wave-7 inception sample

Participants assessed in wave-7 SHARELIFE who had not been assigned to the wave-3 inception sample formed a separate, non-overlapping inception sample. Wave-7 SHARELIFE respondents did not have a comparable same-wave regular health module. Exposure was therefore assessed at wave 7 (2017), wave 8 (2019–2020) was the health landmark, and wave 9 (2021–2022) was the single follow-up assessment. The exposure-complete landmark sample included participants aged at least 50 years from 26 countries.

The two SHARE inception samples were never pooled. Their different measurement occasions, country composition, follow-up periods, and risk-set selection were retained in the analysis and interpretation. SHARE estimates are conditional on completing the relevant SHARELIFE interview, surviving and participating at the next regular health landmark, having the childhood indicators observed, and remaining free of the outcome at that landmark.

### Childhood exposure measures

**Figure 1:**
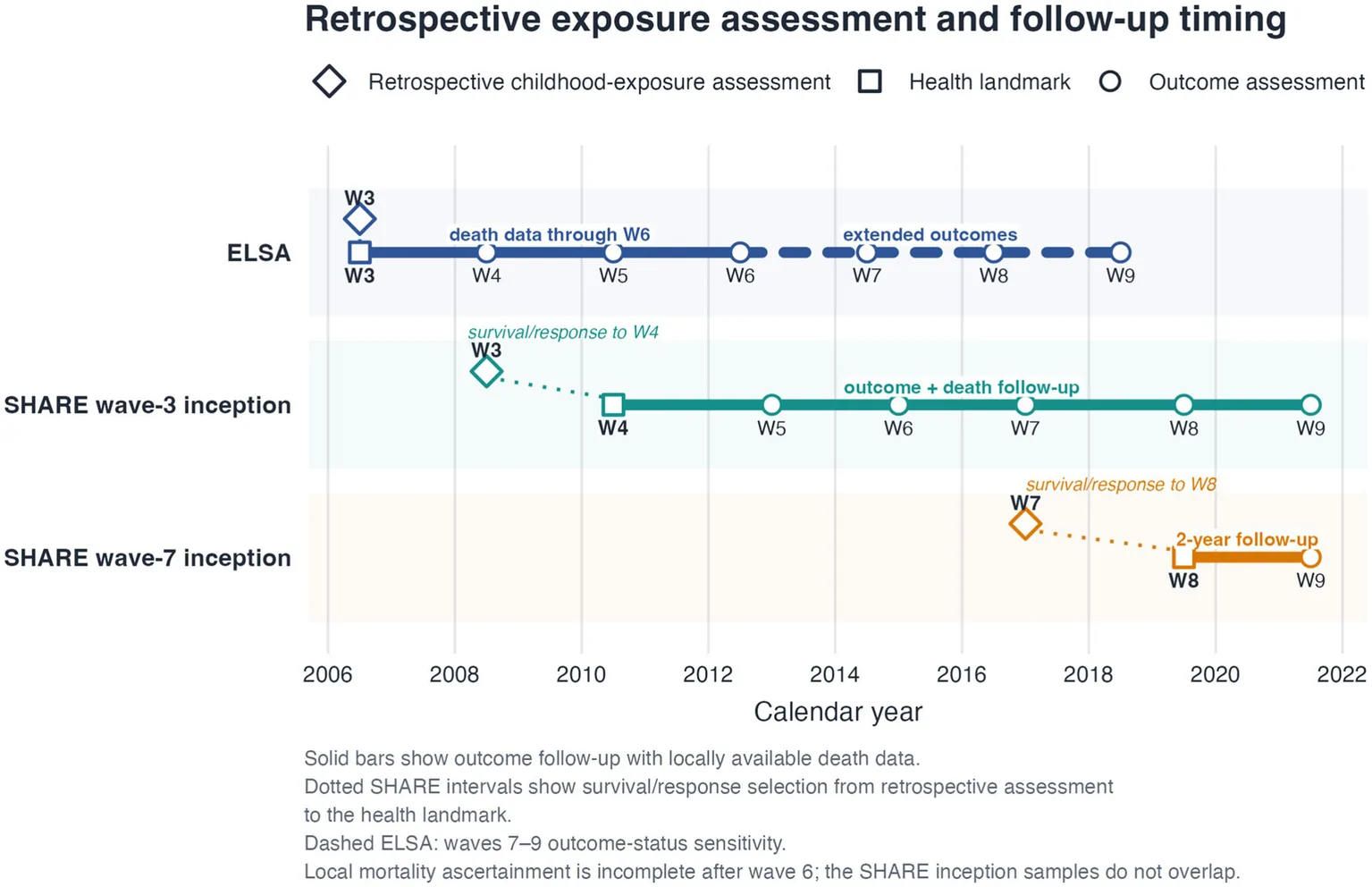
Cohort-specific retrospective exposure assessment, health-landmark, and follow-up timelines. In ELSA, retrospective childhood exposure and baseline health were assessed at wave 3 (2006–2007); waves 4–6 form the locally mortality-ascertained window, and waves 7–9 extend outcome-status follow-up with incomplete mortality ascertainment after wave 6. In the SHARE wave-3 inception sample, retrospective childhood exposure was assessed at wave 3 (2008–2009), baseline health at wave 4 (2010–2011), and outcomes at waves 5–9. In the non-overlapping SHARE wave-7 inception sample, retrospective childhood exposure was assessed at wave 7 (2017), baseline health at wave 8 (2019–2020), and outcomes at wave 9 (2021–2022). Dotted exposure-assessment-to-landmark intervals denote the requirement to survive and participate at the next regular health interview; this pre-landmark selection precedes post-landmark IPCW. The two SHARE inception samples are analyzed separately. The diagram shows scheduled measurement occasions rather than uniform continuous observation.

The common theoretical domain was constrained to early-life health and socioeconomic circumstances that could limit developmental resources. Operational definitions remained cohort-specific. The cohort-specific exposure-assessment, health-landmark, and follow-up timelines are summarized in Figure 1.

#### ELSA childhood-indicator score

One point was assigned for each of the following conditions:

1. Childhood self-rated health reported as fair, poor, or varying greatly;
2. Absence from school for at least 1 month because of health; and
3. Severe financial hardship before age 16.

The childhood financial-hardship variable was used; lifetime financial hardship at any age was not used. The score comprised two health-related indicators and one material-hardship indicator, ranged from 0 to 3, and was analyzed as 0, 1, and at least 2 indicators. The principal contrast was at least 2 versus 0. Because the two health-related indicators can reflect related childhood illness experiences, the score was not interpreted as three independent domains; indicator combinations and indicator-specific models were examined in a sensitivity analysis.

#### SHARE childhood material/cultural-disadvantage score

The SHARE score used only two indicators measured in the same SHARELIFE interview:

1. Lacking at least one of a fixed bath, an indoor toilet, or central heating in the home at age 10; and
2. Having no more than 25 books in the household at age 10.

The score ranged from 0 to 2 and was analyzed as 0, 1, and 2 indicators. The principal contrast compared both material and cultural disadvantages with neither.

Parental education was excluded from the primary SHARE score because it was commonly collected at a later interview. It was examined separately as a supplementary proxy exposure, beginning follow-up only after its actual measurement wave. The ELSA and SHARE exposure categories were not treated as identical levels of a single scale, and their prevalence and effect estimates were not directly ranked. We retained content-defined categories rather than forcing cohort-specific quantiles because the scores had few discrete values and quantile cut points would represent different combinations of domains. Every reported exposure contrast is therefore interpreted within its own cohort and inception sample.

### Outcomes

The outcomes were constructed in both cohorts using the same six ADL activities and seven broad disease groups, drawing on harmonized reports at earlier waves and mapped source items at SHARE wave 9. Source wording and diagnostic context may nevertheless differ between ELSA and SHARE.

ADL-6 disability was difficulty with at least one of six basic activities: walking across a room, dressing, bathing or showering, eating, getting into or out of bed, and toileting. Continence was not included. Any observed positive item classified the participant as having ADL-6 disability; participants with all six items observed and negative classified as free of ADL-6 disability; other item patterns were unclassifiable.

MM-7 multimorbidity was the presence of at least two of seven physician-diagnosed disease groups: hypertension, diabetes, cancer, chronic lung disease, heart disease, stroke, and arthritis. Depression was not included. Once reported, a positive diagnosis was carried forward. When disease items were partly missing, logical bounds were used: a known positive count of at least two was classified as MM-7; a known positive count plus all missing items that kept the count below two was classified as not MM-7; otherwise, the outcome was unclassifiable.

Participants with the relevant outcome at the health landmark were excluded from that outcome-specific risk set. The event was the first observed post-landmark onset. Participants exited the corresponding risk set after first onset, known death, or terminal administrative censoring. The study therefore estimated first observed incidence and did not model recovery after onset as a separate transition.

### Conceptual adjustment strategy

**Figure 2:**
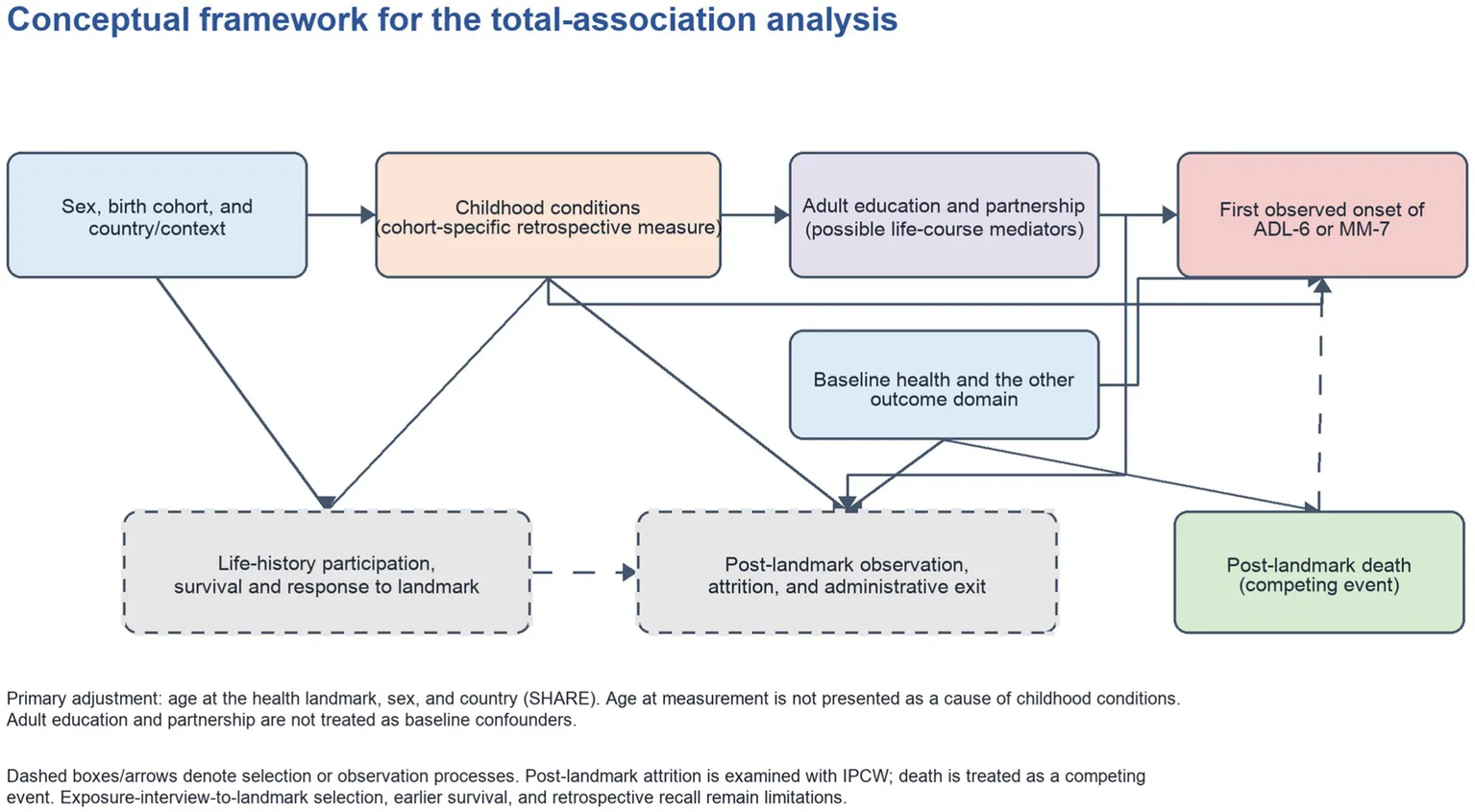
Conceptual framework for the analysis. Cohort-specific adverse or disadvantaged childhood conditions may be associated with late-life ADL-6 disability and MM-7 through health and socioeconomic pathways across adulthood. Adult education and partnership may lie on these pathways and also predict selection into follow-up, as shown by their arrow to post-landmark observation and attrition. Age at the health landmark, sex, and country in SHARE are included in the minimally adjusted association model. Birth cohort, sex, and country/context are distinguished from age at measurement. Life-history participation, survival and response to the health landmark, outcome-free eligibility, post-landmark attrition, administrative exit, and competing death are separate selection processes. Post-landmark attrition is examined using inverse-probability-of-censoring weights (IPCW). The figure is a conceptual framework, not a formal causal directed acyclic graph, and does not assert identification of a causal effect.

The conceptual model distinguished baseline adjustment factors, potential life-course mediators, and selection variables (Figure 2). All outcome models included age at the health landmark and sex. SHARE models also included country fixed effects to account for measured country-level differences in the baseline hazard and exposure/outcome distribution; country fixed effects were not treated as a substitute for the sampling design.

Adult education and marital/partner status were considered potential life-course mediators as well as predictors of follow-up. They were not included automatically in the total-association outcome model. Instead, they entered the denominator of the censoring-weight model. The other baseline health domain—baseline MM-7 status in an ADL-6 analysis and baseline ADL-6 status in an MM-7 analysis—was also included as a selection predictor. We use “association” throughout and do not interpret these models as causal effects.

### Person-period construction and follow-up states

One record represented a scheduled post-landmark assessment interval. Exact valid interview or death month was used when available; otherwise the declared scheduled-wave date was used. Actual interval duration entered each model as an offset. Within-person follow-up time had to be positive and strictly increasing.

Outcome onset and death were mutually exclusive terminal states. In SHARE, Release 9 all-waves coverscreen interview and vital-status codes were linked to each participant-wave. Known death took precedence over non-response. Country-wave non-fieldwork and other documented structural absence were skipped without creating an artificial interval endpoint. Administrative out-of-sample codes ended outcome follow-up at the previous valid endpoint and contributed no artificial event-free person-time. Household/person non-response and item-level outcome missingness were classified as non-structural non-response.

### Survey design and baseline weights

For ELSA, the positive wave-3 life-history respondent weight was used. Government Office Region was treated as the available stratum and household serial as the best locally available cluster; the participant identifier was specified as the final clustering level to account for repeated person-period observations. The original ELSA primary sampling unit was not available locally, so this design is an approximation. Participants missing the available region field were retained in a declared pseudo-stratum.

For SHARE, the exposure-wave-calibrated individual weight was used in the product-weight estimator displayed in the main table: the wave-3-calibrated cross-sectional individual weight for the wave-3 inception sample and the wave-7 SHARELIFE-calibrated individual weight for the wave-7 inception sample. Released weights were retained on their country-specific scale. The pooled coefficient is therefore a weight- and sample-composition-sensitive common-slope summary conditional on country fixed effects; it is neither an equal-country average nor a test of country-specific exposure effects. Official stratum and primary sampling unit identifiers were nested within country. When an official stratum was missing, a country-specific pseudo-stratum was assigned; when an official primary sampling unit was missing, a transparent person-level pseudo-primary sampling unit was used to retain the participant in the design. The participant identifier was specified as the final clustering level to account for repeated person-period observations. Adjacent exposure-to-landmark longitudinal weights were evaluated in the same post-landmark IPCW analysis chain because they additionally condition their calibration on participation and survival across the landmark interval.

The SHARE longitudinal weights represent balanced respondents across the relevant adjacent waves and therefore do not recover people who died or did not participate at the health landmark. Neither weight candidate remains calibrated automatically after exposure completeness, age, and outcome-free restrictions. Accordingly, weighted models are interpreted as design- and selection-sensitive estimates, not guaranteed population-representative estimators.

### Attrition, missing data, and participant selection

The analysis distinguished structural absence from ordinary missingness. Participants who were never administered the relevant life-history module were not treated as having an imputed childhood exposure. Among 7,512 age-eligible ELSA wave-3 landmark respondents, 1,202 had a structurally tagged value indicating that the childhood financial-hardship item was not in their survey when asked; 269 had ordinary item-level incompleteness, and 6,041 had all three indicators observed. Thus, 6,041 of 6,310 respondents for whom all three indicators were in scope were complete (95.7%).

We considered multiple imputation for the 269 ordinarily incomplete ELSA exposure records but did not use it. The retrospective childhood items were the primary exposure rather than nuisance covariates, the missing-at-random assumption was not empirically verifiable, and available later-life variables could make the imputed exposure distribution substantially model-driven. The primary estimand was therefore restricted to exposure-complete respondents; we report their differences from all 1,471 exposure-incomplete landmark respondents and treat the resulting selection as a limitation.

For attrition, we constructed a monotone first-gap analysis. A participant alive and in scope was censored at the first non-structural wave with no classifiable outcome, and later returns were excluded. Stabilized inverse-probability-of-censoring weights (IPCWs) were estimated at each eligible interval. The numerator model included exposure, age, age squared, sex, country in SHARE, and, when more than one scheduled follow-up wave was present, wave. The denominator additionally included adult education, partnership, and the other baseline health domain. Missing optional selection predictors were retained as explicit categories. Cumulative weights were truncated at their analysis-specific first and 99th percentiles. IPCW can reduce selection bias only under its identifying and model-specification assumptions, and it does not address truncation by death unless death is handled separately (20, 21).

The primary outcome-model variables—age, sex, and country where applicable—were complete in all six outcome-specific risk sets; multiple imputation was therefore not required for the primary total-association models. Adult education was missing for approximately 7–8% of the ELSA risk sets and was not made part of the principal outcome model because it was a possible mediator and its missingness was selective. SHARE optional participant covariates were more than 99.7% complete. Survey weights and design variables were not imputed.

**Figure 3:**
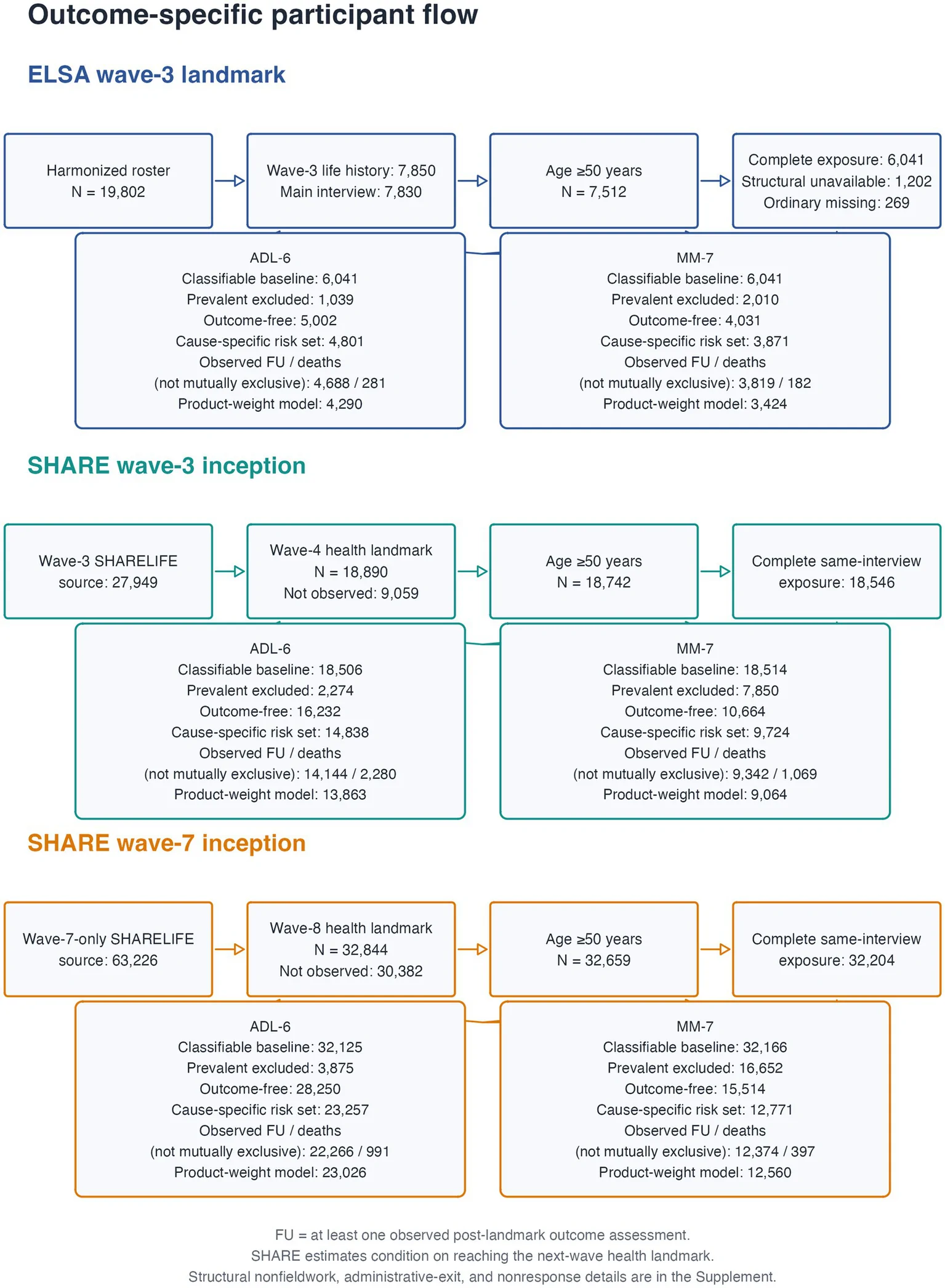
Outcome-specific participant flow for ELSA and the SHARE wave-3 and wave-7 inception samples. The diagram shows source interview, health-landmark participation, age eligibility, complete cohort-specific exposure, classifiable baseline outcome, exclusion of prevalent outcome, observed follow-up or competing death, final cause-specific risk set, and combined weighted/IPCW model inclusion. Observed-follow-up and competing-death counts are not mutually exclusive when an assessment precedes death. Structural non-fieldwork, ordinary non-response, administrative out-of-sample censoring, and known death are reported separately.

Participant selection was described from source interview through health-landmark participation, exposure completeness, outcome-free eligibility, observed follow-up or death, and final model inclusion (Figure 3). For SHARE, participants who reached the next-wave health landmark were compared with those who did not, separately from post-landmark attrition analyses. Included and excluded participants were compared using standardized differences rather than relying only on hypothesis tests.

### Association models

We fitted discrete-time cause-specific hazard models with a complementary log–log link. Models included categorical exposure, baseline age, sex, a flexible wave-specific baseline hazard, and the logarithm of actual interval duration as an offset. SHARE models also included country fixed effects. Survey-design standard errors incorporated the declared multistage design.

This grouped-time approach was chosen because ADL-6 and MM-7 onset was observed only at scheduled interviews and exact onset dates were unavailable. Assigning events to interview dates or interval midpoints in a conventional continuous-time Cox model would introduce artificial timing precision. With a wave-specific baseline hazard and the interval-duration offset, the complementary-log–log model estimates a piecewise-constant cause-specific hazard while retaining unequal interval lengths. A separate death hazard was combined with the outcome hazard to estimate cumulative incidence; a Fine–Gray model would estimate a different sub-distribution-hazard parameter, and a multistate model would address recovery or recurrent transitions rather than the stated first-observed-onset estimand.

We retained the following estimators for triangulation:

1. Unweighted participant-cluster-robust available-follow-up estimates;
2. Baseline survey-weighted estimates;
3. Monotone first-gap estimates with and without stabilized IPCW; and
4. Product-weight estimates using baseline survey weight × truncated IPCW, with participant-cluster-robust and declared available survey-design variance estimates reported in parallel.

For compact reporting, the Results designate the product-weight point estimates with the declared available survey-design variance, interpreted alongside the participant-cluster-robust and other estimators retained in the Supplementary Figure S1. This designation does not imply that the displayed estimator is uniquely valid or population representative. The pseudo-design substitutions are variance approximations rather than a reconstruction of unavailable official design fields, and no single estimator was considered free of assumptions. Hazard ratios (HRs) and 95% confidence intervals (CIs) were reported for exposure category 1 and the cohort-specific highest category versus 0; emphasis was placed on estimates and precision rather than isolated *p*-values.

### Competing-risk standardized absolute effects

Separate discrete-time cause-specific models were fitted for first observed outcome and known death, and their hazards were combined to estimate standardized cumulative incidence functions. Models used the product of baseline survey weight and truncated stabilized IPCW with the declared available survey-design approximation. Risks were standardized to the positive-baseline-weight, outcome-free landmark population by averaging over its baseline age, sex, and country distribution.

We report 6-year risks for the mortality-ascertained ELSA waves 4–6 window and for the SHARE wave-3 sample through wave 7, and 2-year risks for the SHARE wave-7 sample. Risk differences (RDs) are expressed in percentage points and are accompanied by risk ratios (RRs). Uncertainty was estimated using 1,000 multivariate *t* draws from the joint survey-linearized covariance of the outcome and death models. These intervals do not propagate uncertainty from estimation of the IPCW or from the empirical standardization distribution.

### Sensitivity analyses

We examined the extended ELSA wave 4–9 outcome window, available-returner versus monotone first-gap analyses, baseline-weight-only versus combined weighting, the alternative SHARE adjacent-wave longitudinal-weight candidates within the same post-landmark IPCW chain, and two approaches to SHARE administrative censoring. We also described selection between SHARELIFE exposure assessment and the next-wave health landmark. To compare approximately two-year observation windows, we restricted baseline survey-weighted models in each inception sample to the first post-landmark assessment. In the main censoring model, the terminal administrative contact entered as non-response in the observation model but contributed no outcome person-time; in sensitivity analysis, follow-up was independently truncated before that contact. Unplanned final model-form diagnostics added baseline age squared and tested exposure-category-by-wave interactions when at least two follow-up intervals were available.

In total, three bounded closure sensitivities followed those diagnostics. First, because the ELSA ADL-6 interaction suggested possible concentration in wave 4, we required an observed ADL-6-free wave-4 landmark and began follow-up at wave 5. Second, the six standardized absolute-risk analyses were repeated with centered age and age squared in both the outcome and death models. Third, the four SHARE association models and four corresponding absolute-risk analyses were repeated after scaling the released baseline weight within country either to mean 1 or to equal aggregate country weight. For the absolute-risk analyses, each scaling variant was used in both hazard estimation and the baseline standardization distribution. These unplanned analyses did not replace the dated main models or define new primary estimands.

For ELSA, we reported all eight combinations of the three childhood indicators and fitted exploratory indicator-specific models using the same outcome risk sets and product-weight framework. These models were used to examine whether the composite association was concentrated in the two health-related indicators; they were not treated as confirmatory causal decompositions.

Parental education in SHARE was analyzed separately at its true measurement landmark. A parent was classified as having low education if either observed parent had International Standard Classification of Education 1997 level 0–2. This exposure was not reincorporated into the same-interview housing/books score.

Analyses were conducted in R version 4.5.2 using, among other packages, survey version 4.5, survival version 3.8–6, and dplyr version 1.2.0.

## Results

### Participant flow and baseline characteristics

In ELSA, the harmonized roster contained 19,802 participants; 7,850 completed the wave-3 life-history interview, 7,830 also responded to the wave-3 main interview, and 7,512 were aged at least 50 years. Of these age-eligible landmark respondents, 6,041 had all three childhood indicators observed; 1,202 had a structural not-in-survey tag for the childhood financial-hardship item, and 269 had ordinary item-level incompleteness. Exposure completeness was therefore 80.4% of all age-eligible landmark respondents and 95.7% of the 6,310 for whom all three indicators were in scope. The score distribution was 71.7% with 0, 21.0% with 1, and 7.3% with at least 2 indicators. Among the 439 participants in the highest category, 408 (92.9%) had both health-related indicators and 47 (10.7%) had childhood financial hardship; these groups overlap for participants with all three indicators. Mean age was 65.3 years (standard deviation [SD] 10.0), and 55.3% were women. At the landmark, 1,039 of 6,041 participants (17.2%) had ADL-6 disability and 2,010 (33.3%) had MM-7.

After excluding prevalent outcomes, the ELSA risk sets contained 5,002 participants for ADL-6 and 4,031 for MM-7. The final cause-specific risk sets included 4,801 and 3,871 participants, respectively; 4,688 (93.7%) and 3,819 (94.7%) had at least one observed outcome follow-up. Known competing death before outcome occurred in 281 and 182 participants. After the positive baseline-weight and monotone first-gap requirements, the product-weight models included 4,290 participants for ADL-6 and 3,424 for MM-7.

The SHARE wave-3 inception source contained 27,949 SHARELIFE respondents. Of these, 18,890 completed the wave-4 health landmark, 18,742 were aged at least 50 years, and 18,546 had a complete same-interview two-indicator score. Mean age was 68.1 years (SD 9.4), 56.2% were women, and the score distribution was 11.7% with neither disadvantage, 28.2% with one, and 60.1% with both. ADL-6 was present in 2,274 of 18,506 participants with classifiable ADL-6 (12.3%); MM-7 was present in 7,850 of 18,514 with classifiable MM-7 (42.4%).

The wave-3 baseline-free risk sets contained 16,232 participants for ADL-6 and 10,664 for MM-7; 14,838 and 9,724 entered the final cause-specific risk sets. At least one observed outcome follow-up was available for 14,144 (87.1%) and 9,342 (87.6%), and 2,280 and 1,069 participants, respectively, had a known competing death before outcome. The combined weighted/IPCW models included 13,863 participants for ADL-6 and 9,064 for MM-7.

The SHARE wave-7-only source contained 63,226 SHARELIFE respondents. Wave-8 landmark interviews were available for 32,844, of whom 32,659 were aged at least 50 years, and 32,204 had both childhood indicators observed. Mean age was 69.5 years (SD 9.4), 57.7% were women, and 12.7, 30.8, and 56.5% had neither, one, and both disadvantages, respectively. ADL-6 prevalence was 3,875 of 32,125 (12.1%), and MM-7 prevalence was 16,652 of 32,166 (51.8%).

The wave-7 baseline-free risk sets contained 28,250 participants for ADL-6 and 15,514 for MM-7. Final cause-specific risk sets included 23,257 and 12,771; 22,266 (78.8%) and 12,374 (79.8%) had an observed wave-9 outcome assessment, and known competing death occurred in 991 and 397 participants. The product-weight models included 23,026 participants for ADL-6 and 12,560 for MM-7.

Overall, 67.6% of the SHARE wave-3 source and 51.9% of the wave-7-only source reached the next regular health landmark. Landmark participation declined across exposure categories: from 75.9% for score 0 to 64.3% for score 2 in wave 3, and from 55.4 to 50.7% in wave 7. Among those not reaching the landmark, known death or end-of-life status accounted for 11.1% in the wave-3 source and 10.5% in the wave-7 source; the remainder mainly reflected country/batch non-fieldwork, non-participation, or absence of a main interview while known alive. These diagnostics show that the SHARE inception analyses condition on a selected survivor/respondent population (Supplementary Tables S23A–B; Supplementary Figure S3A).

Among participants retained in the product-weight models, median follow-up to the last retained outcome/death interval in the ELSA mortality-ascertained analysis was 6.0 years (interquartile range [IQR] 4.0–6.0). In the extended ELSA window, it was 8.0 years (IQR 4.0–12.0). Corresponding medians in SHARE wave 3 were 6.1 years (IQR 3.6–10.5) for ADL-6 and 5.8 years (IQR 2.3–8.8) for MM-7; in SHARE wave 7 they were 2.1 years (IQR 1.9–2.3) for both outcomes.

Baseline characteristics and exposure distributions are shown in Table 1. Detailed outcome-specific exclusions, country composition, follow-up states, administrative exits, and included-versus-excluded standardized differences are provided in Figure 3 and Supplementary Tables S3 and S5–S11.

**Table 1:** Baseline characteristics of the ELSA wave-3, SHARE wave-3, and SHARE wave-7 exposure-complete landmark samples.

Characteristic	ELSA wave 3	SHARE wave-3 inception	SHARE wave-7 inception
Exposure-complete landmark sample, *N*	6,041	18,546	32,204
Countries represented, *N*	1	12	26
Age, mean (SD), years	65.3 (10.0)	68.1 (9.4)	69.5 (9.4)
Women, *n* (%)	3,339 (55.3%)	10,431 (56.2%)	18,592 (57.7%)
Education: low/primary or less, *n* (%)	1,899 (34.2%)	8,660 (46.7%)	10,225 (31.8%)
Education: middle/secondary, *n* (%)	2,675 (48.2%)	6,190 (33.4%)	14,511 (45.1%)
Education: high/tertiary, *n* (%)	980 (17.6%)	3,696 (19.9%)	7,468 (23.2%)
Education missing, *n*	487	0	0
Married/partnered, *n* (%)	4,380 (72.5%)	14,103 (76.2%)	22,786 (70.8%)
Partnership status missing, *n*	0	42	15
Exposure score 0, *n* (%)	4,333 (71.7%)	2,164 (11.7%)	4,097 (12.7%)
Exposure score 1, *n* (%)	1,269 (21.0%)	5,227 (28.2%)	9,916 (30.8%)
Highest exposure category, *n* (%)	439 (7.3%)	11,155 (60.1%)	18,191 (56.5%)
Baseline ADL-6 disability, *n*/*N* (%)	1,039/6,041 (17.2%)	2,274/18,506 (12.3%)	3,875/32,125 (12.1%)
Baseline ADL-6 unclassifiable, *n*	0	40	79
Baseline MM-7 multimorbidity, *n*/*N* (%)	2,010/6,041 (33.3%)	7,850/18,514 (42.4%)	16,652/32,166 (51.8%)
Baseline MM-7 unclassifiable, *n*	0	32	38

Values are unweighted mean (SD), *n* (%), or *n*/*N* (%) as indicated. Education and partnership percentages use non-missing denominators. ELSA exposure categories are 0, 1, and at least 2 indicators (two health-related and one material-hardship indicator); SHARE categories are neither, one, and both same-interview childhood material/cultural disadvantages. Highest-category prevalence is not directly comparable across cohorts. ADL-6 = difficulty with at least one of six activities of daily living; MM-7 = at least two of seven disease groups.

### Incident ADL-6 disability

In the ELSA mortality-ascertained waves 4–6 analysis, the baseline-weight × IPCW model with declared available survey-design variance contained 10,936 person-intervals, 852 first ADL-6 events, and 215 competing deaths. Compared with score 0, an adversity score of at least 2 was associated with a higher ADL-6 cause-specific hazard (HR 1.43, 95% CI 1.09–1.87). The extended waves 4–9 analysis included 17,435 intervals and 1,252 events; the association was similar (HR 1.47, 95% CI 1.19–1.82), although deaths after wave 6 were incompletely ascertained.

In the SHARE wave-3 inception sample, the ADL-6 model included 41,635 person-intervals, 2,721 events, and 1,735 competing deaths. Score 2 versus 0 was associated with a higher cause-specific hazard (HR 1.36, 95% CI 1.08–1.71). In the wave-7 inception sample, which contributed one follow-up interval per analyzed participant, there were 1,432 ADL-6 events and 990 competing deaths among 23,026 participants; the corresponding HR was 1.60 (95% CI 1.06–2.43).

**Figure 4:**
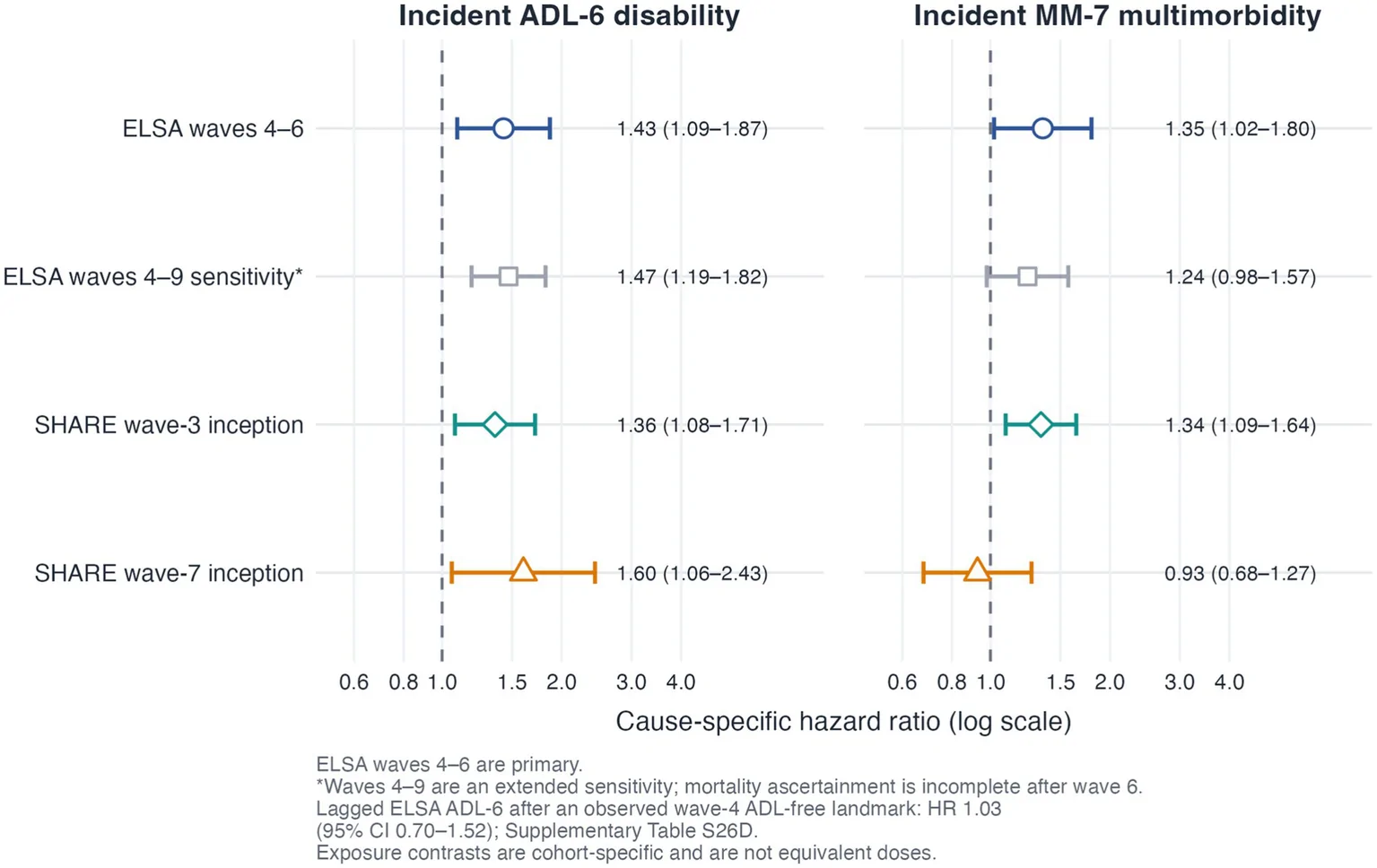
Cohort-specific associations with first observed ADL-6 disability and MM-7 multimorbidity. Points and error bars show hazard ratios (*HRs*) and 95% confidence intervals (*CIs*) for the highest cohort-specific exposure category versus zero from baseline-weight × IPCW complementary log–log models using the declared available survey-design variance approximation. ELSA waves 4–6 are the primary mortality-ascertained estimates; waves 4–9 are displayed as an extended sensitivity with incomplete mortality ascertainment after wave 6. After an observed wave-4 ADL-6-free landmark, the lagged ELSA waves 5–6 *HR* was 1.03 (95% *CI* 0.70–1.52; Supplementary Table S26D). SHARE wave-3 and wave-7 inception samples are not pooled. Vertical line indicates *HR* = 1.0. Effect magnitudes should not be interpreted as direct comparisons of a measurement-invariant exposure.

Thus, the direction of the highest-category ADL-6 contrast was positive in the primary ELSA and both SHARE inception-sample analyses. The intermediate category was not uniformly associated with ADL-6; the lagged ELSA result is reported below, and the category-specific estimates should not be interpreted as a common cross-cohort dose–response gradient (Table 2; Figure 4).

**Table 2:** Cause-specific discrete-time associations between cohort-specific childhood exposure categories and first observed ADL-6 disability or MM-7 multimorbidity.

Sample	Outcome	*N* participants	Person-intervals	Events	Deaths	Contrast	HR (95% CI)	*p* value
ELSA waves 4–6	ADL-6	4,290	10,936	852	215	1 vs. 0	1.05 (0.89–1.25)	0.547
ELSA waves 4–6	ADL-6	4,290	10,936	852	215	≥2 vs. 0	1.43 (1.09–1.87)	0.009
ELSA waves 4–9^†^	ADL-6	4,290	17,435	1,252	215	1 vs. 0	1.03 (0.90–1.18)	0.679
ELSA waves 4–9^†^	ADL-6	4,290	17,435	1,252	215	≥2 vs. 0	1.47 (1.19–1.82)	<0.001
ELSA waves 4–6	MM-7	3,424	8,812	736	145	1 vs. 0	1.00 (0.83–1.20)	0.972
ELSA waves 4–6	MM-7	3,424	8,812	736	145	≥2 vs. 0	1.35 (1.02–1.80)	0.035
ELSA waves 4–9^†^	MM-7	3,424	13,861	1,235	145	1 vs. 0	1.03 (0.89–1.19)	0.704
ELSA waves 4–9^†^	MM-7	3,424	13,861	1,235	145	≥2 vs. 0	1.24 (0.98–1.57)	0.075
SHARE wave-3 inception	ADL-6	13,863	41,635	2,721	1,735	1 vs. 0	1.09 (0.86–1.38)	0.471
SHARE wave-3 inception	ADL-6	13,863	41,635	2,721	1,735	2 vs. 0	1.36 (1.08–1.71)	0.010
SHARE wave-3 inception	MM-7	9,064	24,253	3,536	802	1 vs. 0	1.26 (1.03–1.54)	0.024
SHARE wave-3 inception	MM-7	9,064	24,253	3,536	802	2 vs. 0	1.34 (1.09–1.64)	0.005
SHARE wave-7 inception	ADL-6	23,026	23,026	1,432	990	1 vs. 0	1.36 (0.88–2.09)	0.167
SHARE wave-7 inception	ADL-6	23,026	23,026	1,432	990	2 vs. 0	1.60 (1.06–2.43)	0.026
SHARE wave-7 inception	MM-7	12,560	12,560	1,705	396	1 vs. 0	0.84 (0.62–1.15)	0.279
SHARE wave-7 inception	MM-7	12,560	12,560	1,705	396	2 vs. 0	0.93 (0.68–1.27)	0.639

Hazard ratios use the product of baseline survey weight and stabilized monotone first-gap IPCW, truncated at the first and 99th percentiles, with variance from the declared available survey-design approximation. Models include baseline age, sex, assessment-wave baseline hazard, and actual interval duration; SHARE models additionally include country fixed effects. ELSA waves 4–6 are the locally mortality-ascertained window. ^†^ELSA waves 4–9 have incomplete mortality ascertainment after wave 6. ELSA ≥2 versus 0 and SHARE 2 versus 0 are cohort-specific contrasts, not equivalent exposure doses. ADL-6, activities-of-daily-living disability; CI, confidence interval; HR, hazard ratio; IPCW, inverse-probability-of-censoring weight; MM-7, multimorbidity.

### Incident MM-7 multimorbidity

The ELSA waves 4–6 MM-7 model included 8,812 person-intervals, 736 first MM-7 events, and 145 competing deaths. The HR for an adversity score of at least 2 versus 0 was 1.35 (95% CI 1.02–1.80). Over the extended waves 4–9 window, 1,235 MM-7 events occurred in 13,861 intervals. The estimate was smaller, and its CI included 1 (HR 1.24, 95% CI 0.98–1.57), indicating sensitivity to the observation window.

In SHARE wave 3, the MM-7 model included 24,253 intervals, 3,536 events, and 802 competing deaths. Score 2 versus 0 was associated with a higher MM-7 hazard (HR 1.34, 95% CI 1.09–1.64). In SHARE wave 7, 1,705 events and 396 competing deaths occurred among 12,560 analyzed participants. No clear association was observed; the estimate was imprecise and compatible with no association (HR 0.93, 95% CI 0.68–1.27).

The MM-7 findings therefore did not support a single consistently positive conclusion: the association was positive in the mortality-ascertained ELSA window and SHARE wave 3, weaker in the extended ELSA window, and unclear in SHARE wave 7.

### Standardized risks in the presence of competing mortality

At 6 years in ELSA, standardized ADL-6 risk was 26.9% in the highest ELSA category and 19.8% in score 0, an RD of 7.1 percentage points (95% CI 2.0–12.9) and an RR of 1.36 (95% CI 1.10–1.67). Standardized MM-7 risk was 28.2% versus 21.6%, an RD of 6.6 percentage points (95% CI 0.1–12.9) and an RR of 1.31 (95% CI 1.00–1.62); the lower confidence limits were close to the null.

In SHARE wave 3 at 6 years, standardized ADL-6 risk was 18.9% for score 2 and 14.4% for score 0 (RD 4.5 percentage points, 95% CI 0.7–8.0; RR 1.32, 95% CI 1.04–1.69). MM-7 risk was 36.6% versus 28.6% (RD 8.0 percentage points, 95% CI 2.4–13.1; RR 1.28, 95% CI 1.07–1.52).

**Figure 5:**
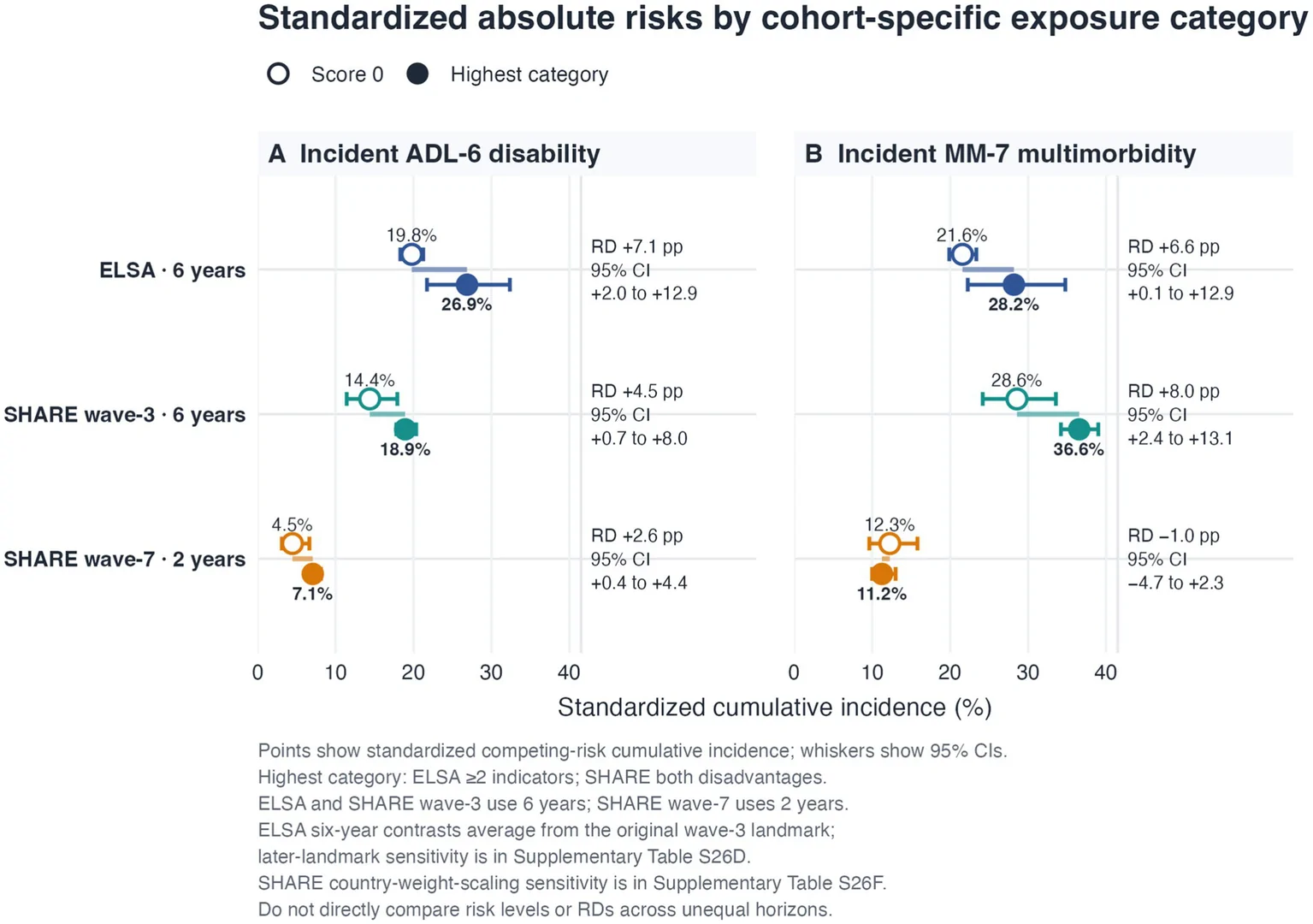
Standardized cumulative incidence in the presence of competing mortality. Paired points and 95% confidence intervals (CIs) show standardized risk for the cohort-specific highest exposure category and zero exposure; connecting lines compare categories only within the same sample, and labels report the corresponding risk difference (RD) in percentage points. ELSA and SHARE wave-3 risks are estimated at 6 years, whereas SHARE wave-7 risks are estimated at 2 years. The ELSA 6-year estimate averages from the original wave-3 landmark; the later-landmark sensitivity is reported in Supplementary Table S26D. The different horizons and cohort-specific exposure definitions preclude direct comparison of absolute-risk magnitude across samples. Estimates use separate cause-specific outcome and death models with the product of baseline survey weight and truncated stabilized inverse-probability-of-censoring weight (IPCW) and declared available survey-design information. SHARE sensitivity to country-weight scaling is reported in Supplementary Table S26F.

In SHARE wave 7 at 2 years, standardized ADL-6 risk was 7.1% for score 2 and 4.5% for score 0 (RD 2.6 percentage points, 95% CI 0.4–4.4; RR 1.58, 95% CI 1.06–2.36). MM-7 risk was 11.2% versus 12.3% (RD –1.0 percentage points (95% CI –4.7 to 2.3); RR 0.92, 95% CI 0.70–1.24). These absolute effects reproduced the distinction between positive ADL-6 contrasts in the primary ELSA analysis and both SHARE inception-sample analyses and heterogeneous MM-7 findings (Table 3; Figure 5). Figure 5 presents the paired standardized risks within each sample; the 6-year ELSA and SHARE wave-3 estimates should not be compared directly with the two-year SHARE wave-7 estimates.

**Table 3:** Standardized cumulative incidence of ADL-6 and MM-7 in the presence of competing mortality.

Sample	Outcome	Horizon	*N* analyzed	Risk: zero exposure (95% CI)	Risk: highest exposure (95% CI)	Risk difference, percentage points (95% CI)	Risk ratio (95% CI)
ELSA	ADL-6	6 years	4,290	19.8% (18.3–21.2)	26.9% (21.7–32.4)	+7.1 (+2.0 to +12.9)	1.36 (1.10–1.67)
ELSA	MM-7	6 years	3,424	21.6% (19.9–23.4)	28.2% (22.3–34.8)	+6.6 (+0.1 to +12.9)	1.31 (1.00–1.62)
SHARE wave-3 inception	ADL-6	6 years	13,863	14.4% (11.4–17.9)	18.9% (17.8–20.3)	+4.5 (+0.7 to +8.0)	1.32 (1.04–1.69)
SHARE wave-3 inception	MM-7	6 years	9,064	28.6% (24.2–33.6)	36.6% (34.2–39.0)	+8.0 (+2.4 to +13.1)	1.28 (1.07–1.52)
SHARE wave-7 inception	ADL-6	2 years	23,026	4.5% (3.1–6.6)	7.1% (6.4–8.1)	+2.6 (+0.4 to +4.4)	1.58 (1.06–2.36)
SHARE wave-7 inception	MM-7	2 years	12,560	12.3% (9.6–15.8)	11.2% (10.0–13.0)	−1.0 (−4.7 to +2.3)	0.92 (0.70–1.24)

Standardized cumulative incidence estimates account for competing mortality using separate cause-specific outcome and death models with the product of baseline survey weight and truncated stabilized IPCW, with the declared available survey-design approximation. Primary models include baseline age as a linear term; adding age squared to both cause-specific models produced materially unchanged contrasts (Supplementary Table S26C). Risks are standardized to the positive-baseline-weight outcome-free landmark population. Risk differences are percentage points. Confidence intervals use 1,000 multivariate t draws from the joint survey-linearized covariance of the outcome and death models and treat IPCW estimation and the empirical standardization distribution as fixed. SHARE country-weight-scaling sensitivity estimates are reported in Supplementary Table S26F. ADL-6, activities-of-daily-living disability; CI, confidence interval; MM-7, multimorbidity.

### Attrition, survey design, and sensitivity analyses

The principal outcome covariates were complete in every risk set. The narrow IPCW distributions produced little additional effective-sample-size loss, and IPCW-only estimates were close to the corresponding first-gap-censored estimates. ELSA baseline weights retained approximately 93% of the positive-weight analytic sample as Kish effective sample size. SHARE baseline weights were more variable: the corresponding effective-sample-size proportions were approximately 38–41% in wave 3 and 24–25% in wave 7, contributing to the wider weighted CIs.

Across alternative estimators applied to the original landmark analyses, available-returner, baseline-weighted, first-gap, IPCW, and product-weight models yielded the same broad interpretation: positive ADL-6 estimates in ELSA and both SHARE inception samples; a positive SHARE wave-3 MM-7 estimate; and no clear SHARE wave-7 MM-7 association. ELSA MM-7 remained dependent on the observation window (Supplementary Figure S1). This estimator triangulation did not assess persistence after excluding wave-4 ADL-6 onset; that question was addressed by the lagged analysis below.

In the approximately 2-year, first-post-landmark survey-weighted comparison, highest-versus-zero ADL-6 HRs were 1.95 (95% CI 1.39–2.73) in ELSA, 1.71 (1.02–2.86) in SHARE wave 3, and 1.61 (1.06–2.43) in SHARE wave 7. The corresponding MM-7 HRs were 1.41 (0.93–2.14), 1.35 (0.99–1.85), and 0.93 (0.68–1.27). This common-window sensitivity retained the distinction between the ADL-6 and MM-7 patterns but was less precise than the longer analyses.

Treating SHARE administrative exits as independent truncation rather than as terminal non-response in the observation model produced nearly identical highest-category HRs: 1.36 versus 1.36 for wave-3 ADL-6, 1.34 versus 1.34 for wave-3 MM-7, 1.60 versus 1.60 for wave-7 ADL-6, and 0.93 versus 0.93 for wave-7 MM-7. In the otherwise identical post-landmark IPCW chain, replacing the SHARE exposure-wave cross-sectional candidate with the adjacent longitudinal candidate changed the highest-category HR by no more than 6.02% and did not change whether its 95% CI included 1. Longitudinal-candidate HRs were 1.35 (95% CI 1.06–1.71) for wave-3 ADL-6, 1.34 (1.09–1.64) for wave-3 MM-7, 1.69 (1.07–2.68) for wave-7 ADL-6, and 0.98 (0.72–1.35) for wave-7 MM-7 (Supplementary Tables S24A–B; Supplementary Figure S3B).

In exploratory ELSA indicator-specific models for the mortality-ascertained waves 4–6 window, adverse childhood self-rated health was associated with ADL-6 (HR 1.29, 95% CI 1.04–1.61) and MM-7 (HR 1.31, 1.04–1.64). The corresponding 6-year RDs were 5.1 percentage points (95% CI 1.0–9.7) and 5.5 percentage points (0.7–10.9). Estimates for prolonged health-related school absence and childhood financial hardship had CIs spanning the null. Mutual adjustment for all three indicators produced similar estimates for childhood self-rated health (ADL-6 HR 1.26, 1.01–1.57; MM-7 HR 1.30, 1.03–1.65). The financial-hardship estimates were imprecise because only 83 exposed participants and 19 exposed events remained in the MM-7 product-weight model. These exploratory results show that the ELSA composite pattern was concentrated in its childhood-health content, but they do not identify an independent causal component (Supplementary Tables S25A–C; Supplementary Figure S2).

When low parental education was analyzed from its true measurement landmark, all survey-weighted CIs included 1: SHARE wave-3 HRs were 1.07 (95% CI 0.89–1.30) for ADL-6 and 1.09 (0.92–1.30) for MM-7; SHARE wave-7 HRs were 1.06 (0.63–1.79) and 1.09 (0.77–1.54), respectively. Irrespective of these estimates, parental education remains a distinct proxy exposure because its construct and measurement time differ from the same-interview material/cultural-disadvantage score.

In unplanned model-form diagnostics, five of eight age-squared terms had *p* < 0.05, indicating age non-linearity, but adding age squared changed the highest-versus-zero HRs by at most 5.5% and did not alter whether any 95% CI included 1. Re-estimating the six standardized absolute-risk contrasts with age squared in both cause-specific models changed RDs by no more than 0.4 percentage points and RRs by no more than 4.9%; no substantive interpretation changed. One of six estimable exposure-category-by-wave tests was significant (ELSA ADL-6 waves 4–6, *p* = 0.019), with possible concentration in the earliest interval; the six-wave waves 4–9 global test was not significant (*p* = 0.120), and the other four tests were not significant. Because these ELSA windows overlap, the longer-window test is not an independent replication. SHARE wave 7 had only one follow-up interval and could not support a time-interaction test (Supplementary Tables S26A–C).

In the resulting lagged ELSA ADL-6 sensitivity, 3,598 participants were observed ADL-6-free at wave 4 and entered the product-weight model. Through waves 5–6, 460 first onsets and 140 competing deaths occurred; the highest-versus-zero HR was 1.03 (95% CI 0.70–1.52). Through waves 5–9, with incomplete mortality ascertainment after wave 6, 860 events occurred and the HR was 1.27 (0.98–1.64). Thus, the primary ELSA ADL-6 association appeared strongest in the earliest wave-4 interval and was attenuated after conditioning on survival, wave-4 response, remaining ADL-6-free, and later observability. The lagged design cannot distinguish temporal attenuation from that of additional landmark selection (Supplementary Table S26D).

Scaling SHARE baseline weights within country changed four of eight diagnostic HR point estimates by at least 10% but did not change whether any 95% CI included 1. For wave-3 ADL-6, the HR changed from 1.36 to 1.20 under both scaling approaches, with CIs still excluding 1. For wave-7 MM-7, the point estimate changed from 0.93 to 1.14 when country means were set to 1 and to 1.22 when aggregate country weights were equalized; both CIs included 1. SHARE wave-3 MM-7 and wave-7 ADL-6 were less sensitive. Absolute effects were also composition-sensitive. The wave-3 ADL-6 RD changed from 4.5 percentage points on the released scale to 2.5–2.7 percentage points under the alternative scales, with intervals that included or approached 0; the wave-3 MM-7 RD remained positive at 5.3–5.9 percentage points. The wave-7 ADL-6 RD remained positive at 2.0–2.1 percentage points, whereas the uncertain wave-7 MM-7 RD changed from −1.0 to +1.3–2.0 percentage points and all intervals included 0. These diagnostics indicate that pooled relative and absolute point estimates can depend on country contribution, without establishing a preferred target population or changing the conclusion that the wave-7 MM-7 contrast was unclear (Supplementary Tables S26E–F).

## Discussion

### Principal findings

The highest cohort-specific exposure category versus zero was associated with incident ADL-6 disability in the primary ELSA and both SHARE inception-sample analyses, in relative-hazard and standardized absolute-risk models. The ELSA association appeared strongest in the earliest post-landmark interval and was not clearly sustained in the later-landmark sensitivity analysis: after excluding ADL-6 onset observed at wave 4 and resetting the landmark among wave-4 survivors/respondents who remained ADL-6-free, the waves 5–6 estimate was imprecise and compatible with no association, and the waves 5–9 estimate was smaller and imprecise. By contrast, the MM-7 results were heterogeneous. A positive association was observed in ELSA through wave 6 and in the SHARE wave-3 inception sample, but the ELSA estimate weakened over the extended window, and no clear association was observed in the SHARE wave 7 inception sample.

The constant exposure coefficient is therefore an interval-averaged cause-specific hazard ratio over the stated window, not evidence of a time-invariant association. The exposure-by-wave analyses were post-review, multiplicity-unadjusted diagnostics, and the overlapping ELSA waves 4–9 test was not an independent replication. Similarly, the later-landmark analysis conditions on additional survival, wave-4 response, remaining ADL-6-free, and later observability. We use these analyses to qualify the original-landmark ELSA finding without claiming either a persistent long-term effect or proven biological attenuation.

These findings provide prospective, timing-specific evidence for later-life functional disability in two large aging studies and show why multimorbidity should not be summarized by a single pooled or uniformly positive conclusion. They do not demonstrate that the ELSA and SHARE measures represent an invariant exposure, rank countries or cohorts by susceptibility, explain cross-cohort heterogeneity, or identify selective survival as its mechanism.

### Interpretation in relation to existing evidence

The ADL-6 findings are compatible with literature linking adverse childhood experiences and socioeconomic disadvantage to poorer adult physical health (1–5) and less favorable ADL trajectories (10). ADL disability may reflect the cumulative consequences of health insults and constrained socioeconomic resources across the life course. The observed primary risk-difference point estimates—approximately 7 percentage points over 6 years in ELSA, 5 percentage points over 6 years in SHARE wave 3, and 3 percentage points over 2 years in SHARE wave 7—were positive within the modeled samples, with the uncertainty shown in Table 3 and Figure 5. However, the lagged ELSA analysis indicates that the positive constant-association summary may have been driven mainly by onset observed during the first post-landmark interval. This may reflect early persistence of childhood health vulnerability, incipient functional decline at wave 3, recall related to current health, or selection induced by the later wave-4 landmark; it does not establish a constant long-term effect. The standardized risks are model estimates, not clinical thresholds, individual risk predictions, or guaranteed population estimates.

Multimorbidity differs from functional disability in ways that may help explain the less consistent findings. Its measured onset depends on diagnosis, condition-specific recall, healthcare contact, the disease list, and the number of conditions already accumulated before the landmark (11). Recent studies and reviews support an association between childhood adversity or disadvantage and adult multimorbidity but also document substantial heterogeneity, variable exposure definitions, and limited certainty (6–9). Baseline MM-7 prevalence was 33.3% in ELSA, 42.4% in SHARE wave 3, and 51.8% in SHARE wave 7. Excluding prevalent multimorbidity therefore selected increasingly restricted outcome-free populations, especially in the later SHARE inception sample. The wave-7 analysis also had only one approximately two-year interval, and its wave-9 follow-up occurred during 2021–2022. COVID-19-period changes in participation, healthcare contact, diagnosis, and functional-health reporting may therefore have contributed to the observed pattern. The imprecise estimate in that sample could reflect a genuinely weaker association, shorter follow-up, conditioning on remaining MM-7-free to a later age, country or period composition, exposure reporting, or residual selection. Its direction also changed under alternative within-country weight scaling, although every interval remained compatible with no association. The present analyses cannot distinguish these explanations.

### Exposure meaning and cross-cohort comparability

The ELSA measure combined the respondent’s childhood health, prolonged health-related school absence, and severe financial hardship before age 16. The first two are distinct questions but related health indicators: 92.9% of the ELSA highest-score category had both, whereas only 10.7% had childhood financial hardship. The SHARE measure represented household amenities and books at age 10. These indicators belong to a broad life-course family of constrained childhood circumstances, but they are not interchangeable. The highest category comprised 7.3% of ELSA respondents and approximately 56–60% of the SHARE inception samples. Thus, “highest versus zero” is a within-cohort contrast, not a common exposure dose, and the ELSA contrast should not be interpreted as a general measure of cumulative cross-domain socioeconomic adversity.

The ELSA health-related indicators may reflect early illness or injury and could be associated with later disability through persistent impairment, developmental disruption, missed education, or subsequent health trajectories. Financial hardship in ELSA and amenities/books in SHARE more directly index constrained material or cultural resources and may operate through nutrition, housing, education, work, and cumulative socioeconomic pathways. Indicator-specific ELSA models were therefore used as exploratory sensitivity analyses, but they do not identify mediation or make the component constructs interchangeable.

This distinction matters for interpreting the HRs. A larger point estimate in one cohort cannot by itself be interpreted as a stronger causal effect. It may instead reflect item content, category thresholds, age and country composition, recall, or selection into the outcome-free risk set. Cross-national studies have repeatedly shown that later-life health distributions vary across social and institutional settings (13, 14), and harmonized resources facilitate comparison without making non-identical source measures interchangeable (15). Measurement and selection must therefore be separated from contextual explanation. Our analyses preserve these alternatives rather than claiming to resolve them.

Parental education illustrates the timing problem. Treating later-measured parental education as if it were available at the earlier SHARELIFE wave would misalign exposure ascertainment and time zero. Starting follow-up only after the actual parent-education measurement produced weighted estimates with CIs that included 1 for both outcomes. These results do not show that parental education is unimportant; the measured subset was selected, and some estimates were imprecise. They show that parental education should be treated as a distinct proxy exposure with its own time zero.

### Attrition, mortality, and selection

The models incorporated observed follow-up timing, first non-structural gaps, known deaths, structural country-wave absence, and administrative exits. This directly addresses differential observation after the landmark. It does not eliminate selection. In SHARE, substantial selection also occurred between the SHARELIFE exposure interview and the next regular health landmark, before post-landmark IPCW began. Adjacent-wave longitudinal weights and reached-versus-not-reached diagnostics assess this stage only under their assumptions; they cannot recover outcomes for people who died or did not participate at the landmark. Attrition in longitudinal aging studies can itself be associated with mortality (22). IPCW depends on correct models and on follow-up being conditionally independent of selection after the measured predictors. Stability across weighting and censoring approaches cannot demonstrate that selection is ignorable.

Similarly, modeling death after the health landmark addresses competing mortality during observed follow-up but not survival to cohort entry or to the life-history interview. Frailty selection can alter associations among survivors (23), and socioeconomic position is related to premature mortality (24). Selective survival therefore remains one plausible explanation for between-sample differences, alongside age-period-cohort composition (25), recall, study-entry selection, diagnostic opportunity, and attrition. Evidence from older population samples also cautions that retrospective childhood-adversity patterns may reflect both recall and selection processes (17). We did not identify the contribution of any one mechanism.

The ELSA flow also changes the interpretation of missing childhood data. Complete exposure was available for 6,041 of 7,512 age-eligible wave-3 landmark respondents (80.4%); 1,202 had structural item unavailability, and 269 had ordinary item-level incompleteness. Among the 6,310 respondents for whom all three exposure indicators were in scope, completeness was 95.7%. Using the full harmonized enrolled roster as the denominator would yield 32.7%, but that denominator combines structural non-administration with ordinary item-level missingness. Structural non-ascertainment was not imputed, and the ordinary incomplete records were not imputed for the reasons stated in the Methods. Comparisons of complete and incomplete participants remain necessary because this restriction may still induce selection.

### Strengths and limitations

Strengths include the use of two large longitudinal aging studies, separate SHARE inception samples with defensible time zero, incident rather than last-wave outcome status, repeated assessments up to first onset, actual interval-duration offsets, explicit competing mortality, survey-design and attrition diagnostics, standardized absolute effects, and a raw-coverscreen audit of SHARE deaths and fieldwork states. A dated revision-stage analysis plan and amendment trail distinguish these analyses from prospectively pre-specified work.

Several limitations should temper interpretation.

First, childhood conditions were reported retrospectively. Prospective and retrospective childhood-maltreatment measures identify substantially different groups, and retrospective reports in older cohorts may reflect recall, cohort, and selection processes (16, 17). The ELSA and SHARE measures did not undergo a formal measurement-invariance analysis. The broad label “early-life adversity/disadvantage” therefore denotes a conceptual family rather than an item-identical scale. The ELSA analysis also excluded 269 respondents with ordinary item-level exposure incompleteness in addition to 1,202 with structural item unavailability. Multiple imputation would have required an unverifiable missing-at-random assumption for the primary retrospective exposure; restriction to complete exposure may nevertheless bias the estimates, as suggested by observed differences between complete and incomplete groups.

Second, outcomes were based on self-report and harmonized disease groups. Diagnostic practices and healthcare access may differ across countries. MM-7 used seven common disease groups and excluded depression; conclusions may differ with other multimorbidity definitions.

Third, the event was first observed onset between interviews. Exact onset was interval-censored, and the analysis did not model subsequent recovery or recurrent transition. The chosen estimand uses repeated observations to improve timing over a last-wave analysis but is not a full multistate description of disability. The main models summarized a constant exposure association across intervals. An unplanned diagnostic and the subsequent lagged analysis both suggested that the ELSA ADL-6 association was strongest in the first interval. The lagged model nevertheless conditions on an observed wave-4 survivor/respondent who remained ADL-6-free and later observable, so it cannot by itself distinguish true temporal attenuation from selection or an altered risk population.

Fourth, ELSA mortality ascertainment was locally complete only through wave 6. The wave 4–9 models provide extended outcome-status sensitivity estimates, not complete long-term competing-risk estimates. The SHARE wave-7 sample had only one follow-up wave, limiting precision and the ability to distinguish short-term from longer-term associations.

Fifth, survey inference was constrained by locally available design information. The original ELSA primary sampling unit was unavailable and some SHARE observations required pseudo-design substitutions. Official documentation established the general target of the SHARE weight candidates, but the weights were not recalibrated after exposure-complete and outcome-free restrictions; calibration to the final restricted analytic populations therefore remains uncertain. The displayed pooled SHARE coefficient and standardized risks also depend on how country-specific weight scales contribute to model fitting and standardization. Country-rescaling diagnostics preserved the main uncertainty classifications but changed several relative and absolute point estimates, including the direction of the uncertain wave-7 MM-7 contrasts. The product-weight results are therefore design- and selection-sensitive model estimates within a triangulation of estimators, not guaranteed to represent population-level effects. Variable SHARE weights also reduced effective sample size substantially.

Sixth, IPCW estimates depend on measured predictors and correctly specified observation models. The absolute-risk intervals propagated the joint survey-linearized uncertainty of the outcome and death models but treated the estimated IPCW and empirical standardization distribution as fixed.

Finally, residual confounding, exposure measurement error, cohort-entry selection, and survival before observation remain. No sensitivity analysis or statistical adjustment eliminates these threats.

### Public health and research implications

The findings support incorporating early-life conditions into life-course research on later-life functional health. They do not establish a screening instrument for comprehensive geriatric assessment, an individual prediction rule, or a policy-targeting threshold. The practical implication is methodological: international aging studies should align childhood constructs, record when each component was obtained, establish a health landmark at or after exposure ascertainment without back-projecting information collected later, preserve repeated outcome and mortality information, and report absolute as well as relative measures.

Future comparative research would benefit from prospectively measured or carefully validated childhood indicators, formal harmonization work, longer comparable follow-up for later inception samples, and designs capable of separating cohort entry, attrition, mortality selection, and contextual mechanisms. Policies addressing wider social determinants of health remain important (26), but the present observational estimates do not identify which intervention or institutional mechanism would change the observed associations.

## Conclusion

The highest cohort-specific exposure category versus zero was prospectively associated with first observed ADL-6 disability in the primary ELSA and two separately timed SHARE inception-sample analyses. In ELSA, the association appeared strongest in the earliest post-landmark interval and was not clearly sustained after that onset period was excluded. Associations with MM-7 multimorbidity were less consistent: they varied by ELSA observation window, and no clear association was observed in the later SHARE inception sample. These findings support a life-course perspective on late-life functional health while underscoring that exposure non-equivalence, follow-up structure, weighting, attrition, and selection constrain cross-cohort interpretation. The study does not explain the observed heterogeneity or establish selective survival as a mechanism.

## Data Availability

The analyses used registered-access third-party data from the English Longitudinal Study of Ageing (ELSA) and the Survey of Health, Ageing and Retirement in Europe (SHARE). ELSA data are available to registered UK Data Service users at https://beta.ukdataservice.ac.uk/datacatalogue/studies/study?id=5050. SHARE data are available to individually registered users through the SHARE Research Data Center at https://share-eric.eu/data/ become-a-user, subject to the respective conditions of use. The authors cannot redistribute these third-party microdata. Analysis code is available from the corresponding authors on reasonable request.
