# Supplementary material for: Childhood adversity and disadvantage in relation to incident disability and multimorbidity: prospective analyses of ELSA and two SHARE inception samples
Source: Front Public Health. 2026 Sep 8;14:1889565. doi: 10.3389/fpubh.2026.1889565 (PMC13593477; doi:10.3389/fpubh.2026.1889565)
Supplement: Supplementary file 1 [file Table_1.DOCX]

Supplementary Material

### Scope of this supplement

This supplement documents the revision-stage reanalysis of the English Longitudinal Study of Ageing (ELSA) and the Survey of Health, Ageing and Retirement in Europe (SHARE). The initial revision-stage analysis plan was dated 23 July 2026, followed by dated amendments and explicitly unplanned closure diagnostics on 25 and 27 July 2026; none was prospectively pre-specified. The scientific focus is the prospective, cohort-specific association between childhood conditions and first observed onset of activities-of-daily-living limitation or multimorbidity. The childhood measures are not assumed to be item-equivalent across ELSA and SHARE, and their coefficients are not used to rank countries or cohorts.

The supplement provides the detailed definitions, sample construction, survey-design decisions, missing-data assessment, attrition models, estimator comparisons, competing-risk absolute risks, and restricted sensitivity analyses that support the revised manuscript. Historical seven-cohort comparisons, welfare-regime analyses, social-expenditure correlations, E-values, and extensive subgroup analyses have been removed from the revised evidential package.

Three supplementary figures summarize analyses already reported in the frozen tables: estimator triangulation (Figure S1), ELSA indicator-specific results and high-score composition (Figure S2), and SHARE selection between exposure reporting and the next health landmark together with the adjacent-wave longitudinal-weight sensitivity (Figure S3). These figures do not introduce new models or restore analyses removed from the revised evidence.

All numerical values were imported from output files frozen after the corresponding analyses were completed. Document preparation itself did not re-estimate the models.

## Supplementary Methods

### 1. Analysis hierarchy, cohorts, and time zero

ELSA and SHARE were analysed separately. The childhood conditions predated each health landmark, but exposure reporting occurred at the same wave as the health landmark in ELSA and before the landmark in SHARE. The two SHARE inception samples were kept separate because participants who received SHARELIFE in wave 7 did not receive a comparable regular physical-health module in the same wave.

**Supplementary Table S1. Cohort-specific analysis structure**

| **Cohort/source** | **Childhood-exposure interview** | **Health landmark** | **Eligibility at health landmark** | **Follow-up used for association models** | **Mortality qualification** |
| --- | --- | --- | --- | --- | --- |
| ELSA | Wave 3 life-history interview | Wave 3 | Life-history respondent; main-interview respondent; age ≥50 years; complete ELSA exposure; free of the outcome | Waves 4–6 and waves 4–9 | Waves 4–6 are the locally mortality-ascertained window; death ascertainment is incomplete after wave 6 in the locally available data |
| SHARE-W3 | Wave 3 SHARELIFE | Wave 4 regular health interview | Wave-4 respondent; age ≥50 years; complete two-indicator W3 exposure; free of the outcome | Waves 5–9, subject to country-specific fieldwork | Raw all-waves coverscreen states were used to identify death, nonfieldwork, administrative censoring, and ordinary nonresponse |
| SHARE-W7 | Wave 7 SHARELIFE among participants without an earlier W3 SHARELIFE interview | Wave 8 regular health interview | Wave-8 respondent; age ≥50 years; complete two-indicator W7 exposure; free of the outcome | Wave 9 | Raw all-waves coverscreen states were used; this is a single post-landmark assessment window |

*Provenance: `00_protocol/Revision_SAP_v2_2026-07-23.md` and `00_protocol/SAP_amendment_02_SHARE_raw_coverscreen_states_2026-07-23.md`.*

For SHARE, exact valid interview and death months were used when they preserved post-landmark chronology. A declared scheduled-wave date was used only when an exact valid date was unavailable. For ELSA, scheduled two-year wave intervals were used. All retained analytic intervals were required to have positive duration and strictly increasing within-participant analysis time.

### 2. Childhood exposure definitions and comparability

### ELSA

The ELSA score assigned one point for each of the following:

1. self-rated childhood health reported as fair, poor, or having varied greatly;

2. having missed school for at least one month because of health;

3. severe financial hardship before age 16, measured by rasfnhch.

The lifetime financial-hardship variable rasfnhe was not used. The total score ranged from 0 to 3 and was represented categorically as 0, 1, or at least 2 indicators. It contains two health-related indicators and one material-hardship indicator and is not interpreted as three independent domains or as a universal adversity threshold.

### SHARE

The primary SHARE score used two indicators obtained in the same SHARELIFE interview:

1. housing disadvantage at age 10, defined as lacking at least one of a fixed bath, indoor toilet, or central heating; and

2. limited cultural resources at age 10, defined as having no more than 25 books in the household.

The score ranged from 0 to 2 and was represented as neither, one, or both disadvantages. Parental education was excluded from this primary score because the raw questions were commonly administered at a later interview. It was analysed separately from its true measurement landmark.

**Supplementary Table S2. Exposure content and distribution in eligible, exposure-complete health-landmark samples**

| **Cohort/source** | **Common construct used in this study** | **Indicators** | **Categories** | **Category 0, n (%)** | **Category 1, n (%)** | **Highest category, n (%)** |
| --- | --- | --- | --- | --- | --- | --- |
| ELSA-W3 | Cohort-specific adverse childhood health and material conditions | Childhood self-rated health; prolonged school absence for health; severe financial hardship before age 16 | 0, 1, ≥2 | 4,333 (71.73) | 1,269 (21.01) | 439 (7.27) |
| SHARE-W3 | Cohort-specific childhood material and cultural disadvantage | Inadequate housing facilities; ≤25 books at age 10 | 0, 1, 2 | 2,164 (11.67) | 5,227 (28.18) | 11,155 (60.15) |
| SHARE-W7 | Cohort-specific childhood material and cultural disadvantage | Inadequate housing facilities; ≤25 books at age 10 | 0, 1, 2 | 4,097 (12.72) | 9,916 (30.79) | 18,191 (56.49) |

The ELSA highest category combines scores 2 and 3: 423 participants scored 2 and 16 scored 3. Of these 439 participants, 408 (92.9%) had both health-related indicators and 47 (10.7%) had childhood financial hardship; the counts overlap for participants with all three indicators. The large difference between the ELSA and SHARE highest-category prevalences demonstrates why the numerical cut points cannot be interpreted as measurement-equivalent levels of childhood adversity or disadvantage.

*Provenance: `03_results/elsa_exposure_distribution.csv`, `03_results/share_exposure_distribution.csv`, and `03_results/elsa_component_combination_distribution.csv`. The combined ELSA ≥2 count is the arithmetic sum of the frozen score-2 and score-3 rows.*

### 3. SHARE country and wave coverage

The W3 inception sample included 12 countries. The W7-only inception sample included 26 countries; 27 distinct countries were represented across the two inception samples. Country composition differed substantially between inception samples and was therefore not pooled into a single exposure cohort.

**Supplementary Table S3. SHARE inception-sample composition by country**

| **Country** | **W3 inception N** | **W7-only inception N** |
| --- | --- | --- |
| Austria | 705 | 1,236 |
| Germany | 1,420 | 2,153 |
| Sweden | 1,371 | 1,389 |
| Netherlands | 1,694 | — |
| Spain | 1,681 | 1,274 |
| Italy | 2,022 | 1,203 |
| France | 1,880 | 1,416 |
| Denmark | 1,671 | 1,171 |
| Switzerland | 1,109 | 1,196 |
| Belgium | 2,178 | 1,232 |
| Czech Republic | 1,244 | 1,837 |
| Poland | 1,571 | 1,372 |
| Greece | — | 768 |
| Israel | — | 748 |
| Luxembourg | — | 834 |
| Hungary | — | 664 |
| Slovenia | — | 2,270 |
| Estonia | — | 2,814 |
| Croatia | — | 1,133 |
| Lithuania | — | 1,344 |
| Bulgaria | — | 878 |
| Cyprus | — | 498 |
| Finland | — | 1,113 |
| Latvia | — | 722 |
| Malta | — | 765 |
| Romania | — | 1,208 |
| Slovakia | — | 966 |
| Total | 18,546 | 32,204 |

The raw Release 9 coverscreen audit identified three post-landmark country-wave cells without main fieldwork in the W3 risk sets: Poland at wave 5 and the Netherlands at waves 6 and 7. These structural cells were removed from response and outcome calendars without censoring surviving participants. No whole-country nonfieldwork cell occurred between the W7 health landmark and wave 9.

*Provenance: `03_results/share_inception_country_composition.csv`, `03_results/share_release_country_wave_fieldwork_audit.csv`, and `00_protocol/share_structural_fieldwork_weight_audit_2026-07-23.md`.*

### 4. Outcome definitions

### ADL-6

ADL-6 comprised difficulty with walking across a room, dressing, bathing or showering, eating, getting in or out of bed, and toileting. Continence was not included. At each wave:

- any observed positive item classified ADL-6 as positive;
- all six items observed and negative classified ADL-6 as negative;
- all other patterns were unclassifiable.

### MM-7

MM-7 was defined as at least two of seven physician-diagnosed disease groups: hypertension, diabetes, cancer, chronic lung disease, heart disease, stroke, and arthritis. Depression was not included.

**Supplementary Table S4. Cohort-specific outcome construction**

| **Outcome component** | **ELSA waves 3–9** | **SHARE waves 4–8** | **SHARE wave 9** |
| --- | --- | --- | --- |
| Walking across a room | Harmonized walkra binary item | Harmonized walkra binary item | Physical-health item ph049d2 |
| Dressing | Harmonized dressa binary item | Harmonized dressa binary item | ph049d1 |
| Bathing/showering | Harmonized batha binary item | Harmonized batha binary item | ph049d3 |
| Eating | Harmonized eata binary item | Harmonized eata binary item | ph049d4 |
| Getting in/out of bed | Harmonized beda binary item | Harmonized beda binary item | ph049d5 |
| Toileting | Harmonized toilta binary item | Harmonized toilta binary item | ph049d6 |
| Hypertension | Harmonized hibpe, ever diagnosed | Harmonized hibpe, ever diagnosed | ph006d2 |
| Diabetes | Harmonized diabe, ever diagnosed | Harmonized diabe, ever diagnosed | ph006d5 |
| Cancer | Harmonized cancre, ever diagnosed | Harmonized cancre, ever diagnosed | ph006d10 |
| Chronic lung disease | Harmonized lunge, ever diagnosed | Harmonized lunge, ever diagnosed | ph006d6 |
| Heart disease | Harmonized hearte, ever diagnosed | Harmonized hearte, ever diagnosed | ph006d1 |
| Stroke | Harmonized stroke, ever diagnosed | Harmonized stroke, ever diagnosed | ph006d4 |
| Arthritis | Harmonized arthre, ever diagnosed | Harmonized arthre, ever diagnosed | Positive if ph006d19 or ph006d20 was positive |

Once a chronic disease had been reported, the positive status was carried forward so that a later denial or missing value did not create an artificial recovery. At each wave, bounds were used to classify MM-7: a known-positive count of at least 2 was positive; a known-positive count plus the number missing below 2 was negative; otherwise the outcome was unclassifiable.

*Provenance: `00_protocol/Revision_SAP_v2_2026-07-23.md`, `01_scripts/01_build_elsa_landmark.R`, and `01_scripts/02_build_share_landmarks.R`.*

### 5. Person-period construction, event onset, and follow-up states

One record represented a scheduled post-landmark interval. Participants left the risk set at the first observed outcome or known death. Outcome and death were mutually exclusive terminal states. The estimand was first observed onset, so recovery after onset was not modelled.

The available-follow-up estimator permitted a participant to return after a missed assessment; the next observed record spanned the elapsed interval from the last valid endpoint. The monotone estimator instead censored a surviving participant at the first eligible missing outcome assessment and discarded later returns. These approaches answer the same first-onset question under different assumptions about intermittent missingness.

For SHARE, the raw all-waves coverscreen status took precedence over the harmonized interview-status field. States were assigned in the following order:

1. known death;

2. structural nonfieldwork or inactive sample batch;

3. administrative out-of-sample censoring;

4. not-yet-sample diagnostic status;

5. ordinary household or person nonresponse;

6. item missingness at a main interview.

Death overrode all missingness states. A structural skipped wave contributed neither an outcome endpoint nor an IPCW response record, and a later observed assessment could span the structural gap. Administrative censoring ended outcome follow-up at the previous valid endpoint and contributed no artificial event-free person-time.

The raw-state audit identified 174 ADL-6 and 101 MM-7 participants in the W3 inception risk sets whose raw vital-death row had not been terminal in the earlier derived risk set. After giving earlier administrative exit precedence where applicable, 158 and 93 deaths, respectively, moved one scheduled wave earlier in the corrected risk sets. The corrected raw state was applied before the final models were fitted.

### 6. Survey design and baseline weights

ELSA analyses used the positive wave-3 life-history respondent weight (r3lhwtresp). Government Office Region was used as the stratum, household serial as the best locally available cluster, and participant as the repeated-observation stage. The original ELSA primary sampling unit (PSU) was unavailable; the variance design is therefore an approximation and must not be interpreted as a reconstruction of the original design. Participants missing the available region field were retained in a declared pseudo-stratum.

SHARE analyses used the exposure-wave calibrated individual weight (cciw_w3 or cciw_w7_SHL) on its released country-specific scale. The pooled coefficient is therefore a weight- and sample-composition-sensitive common-slope summary conditional on country fixed effects; it is not an equal-country average or a test of country-specific exposure effects. Official stratum and primary-sampling-unit identifiers were nested within country. Where an official stratum code was absent, a country-specific pseudo-stratum was assigned. Where an official primary-sampling-unit code was absent, the participant was used as a transparent ultimate pseudo-primary-sampling unit within country and stratum. These substitutions retained otherwise analysable records but did not reconstruct unknown official design fields.

Adjacent exposure-to-health-landmark longitudinal weights (cliw_d for W3-to-W4 and cliw_h for W7-to-W8) were examined in the same post-landmark IPCW chain as the cross-sectional candidate. The longitudinal weights apply to balanced respondents across the relevant wave pair and target the start-wave age-eligible population surviving to the later wave. They cannot recover participants who died or did not respond at the health landmark, and neither candidate remains automatically calibrated after the study’s exposure-complete and outcome-free restrictions. The two candidates are therefore used for triangulation rather than described as bias-free population-representative estimators.

### 7. Attrition and monotone first-gap IPCW

The attrition analysis used stabilized inverse probability-of-censoring weights (IPCW) on a monotone first-gap calendar. A surviving participant was censored at the first eligible scheduled assessment with an unobserved outcome; observations after that first gap were not allowed to re-enter.

For each eligible response record, the numerator model included the categorical exposure, centred baseline age, squared centred baseline age, sex, scheduled wave when more than one follow-up wave was present, and country in SHARE. The denominator additionally included adult education, partnership status, and the other baseline health domain (baseline MM-7 for the ADL-6 analysis or baseline ADL-6 for the MM-7 analysis). Missing optional selection predictors were retained as explicit categories. Predicted response probabilities were bounded at 0.01 and 0.99. The cumulative stabilized weight was the product of numerator-to-denominator probability ratios over observed responses. Death contributed a factor of 1 and was not treated as nonresponse.

Raw cumulative IPCW values were truncated at analysis-specific 1st and 99th percentiles. For the combined estimator, the truncated IPCW was multiplied by the positive baseline survey weight. The primary SHARE handling retained a terminal administrative-censor record as R=0 in the response model while excluding it from outcome person-time. A declared sensitivity treated this state as independent administrative truncation and omitted its R=0 factor.

IPCW depends on the measured selection model and cannot correct unmeasured selection, exposure measurement error, or an unavailable survey-design field. The product-weight estimator is designated for compact reporting within the triangulation of estimators; its estimates are design- and selection-sensitive model results rather than bias-free population effects.

### 8. Association models and estimands

The cause-specific outcome hazard was estimated using a complementary-log-log model with the logarithm of the actual interval duration as an offset. All models included categorical exposure, baseline age, sex, and a flexible scheduled-wave baseline hazard. SHARE models also included country fixed effects. Repeated observations were handled by participant clustering within the declared survey design.

The grouped-time model was selected because outcome onset was observed only at scheduled interviews and exact onset dates were unavailable. A conventional Cox model would require assigning an artificial event date or midpoint. The complementary-log-log specification instead estimates a piecewise-constant cause-specific hazard over each observed interval while retaining unequal interval lengths. Separate outcome and death hazards were combined for cumulative-incidence estimation; Fine–Gray subdistribution hazards and multistate recovery/recurrent-transition estimands were outside the stated first-observed-onset question.

Adult education and partnership were treated as possible life-course mediators or selection predictors, not automatic confounders in the total-association model. The reported hazard ratio is an association measure for the cause-specific discrete-time hazard and is not a causal effect.

Six estimators were retained for transparent comparison:

1. unweighted, participant-cluster-robust available-follow-up analysis;

2. baseline survey-weighted available-follow-up analysis;

3. unweighted monotone first-gap analysis;

4. unweighted monotone first-gap analysis with stabilized IPCW;

5. baseline survey weight multiplied by IPCW with participant-cluster-robust variance;

6. the same product weight with the declared multistage survey variance design.

During final closure, we added two explicitly unplanned model-form diagnostics to the frozen product-weight analysis. First, baseline centred age squared was added to each of the eight Table-2 models. Second, exposure-category-by-scheduled-wave interactions were tested jointly when an analysis contained at least two scheduled follow-up intervals. Four further bounded sensitivities addressed the resulting interpretive questions: an ELSA ADL-6 analysis beginning after an observed ADL-6-free wave-4 landmark; standardized absolute risks with centred age squared in both cause-specific models; SHARE association models with country-mean-one or equal-country-total baseline-weight scaling; and corresponding SHARE absolute-risk analyses using each scale in both model fitting and standardization. These checks did not replace the dated main model or define a new primary estimand.

### 9. Competing mortality and standardized absolute risk

Separate cause-specific complementary-log-log models were fitted for first observed outcome and known death. The hazards were combined to obtain standardized cumulative incidence functions.

The ELSA absolute-risk analysis used the locally mortality-ascertained waves 4–6 window and reported 2- and 6-year risks. SHARE-W3 used waves 5–7 to define a common 6-year absolute-risk window; this is intentionally shorter than the waves 5–9 cause-specific hazard analysis. SHARE-W7 used wave 9 and reported 2-year risk.

Hazard models used the baseline survey weight multiplied by truncated stabilized IPCW. Cumulative incidence was standardized to the positive-baseline-weight, outcome-free health-landmark risk set and marginalized using normalized baseline survey weights. IPCW entered hazard estimation but not the baseline standardization distribution.

The joint covariance of the outcome and death hazard coefficients was obtained from the multistage survey linearization while preserving cross-cause covariance. One thousand multivariate *t* draws, with analysis-specific survey-design degrees of freedom, produced percentile intervals. These intervals propagated the declared survey variance of the two coefficient vectors. They did not propagate uncertainty from fitting the IPCW models, uncertainty in the empirical standardization distribution, reconstruction of unavailable design fields, or exposure and outcome measurement.

### 10. Baseline missing data and multiple-imputation decision

Age, sex, and country where applicable were complete in all six outcome-specific primary risk sets. Multiple imputation was therefore not needed for the primary outcome-model covariates.

ELSA exposure missingness required a separate decision. Among 7,512 age-eligible wave-3 landmark respondents, 1,202 had the Harmonized ELSA tagged value .a (“not in survey when asked”) for the childhood financial-hardship item. These records were classified as structural item unavailability rather than ordinary missingness. A further 269 respondents had ordinary incompleteness in at least one of the three indicators, and 6,041 had all three observed. Among the 6,310 respondents for whom all three indicators were in scope, 95.7% were complete and 4.3% were ordinarily incomplete.

We considered multiple imputation for the 269 ordinarily incomplete exposure records but did not undertake it because the retrospective items constituted the primary exposure, the missing-at-random assumption could not be evaluated, and imputation from later-life variables could make the reconstructed childhood exposure substantially dependent on model specification. The primary target was therefore restricted to respondents with complete observed exposure. This decision does not eliminate selection bias; complete and incomplete groups are compared in Supplementary Table S10B.

Adult education was missing for approximately 7%–8% of the ELSA outcome-specific samples and was selective with respect to age and sex. Because adult education was treated as a possible mediator rather than a required primary confounder, an education-complete-case model was not substituted mechanically for the primary model. SHARE participant-level optional covariates were nearly complete. Missing survey strata or primary-sampling-unit identifiers were design properties and were not imputed.

### 11. SHARE parental education at the true measurement landmark

Parental education was measured through raw mother and father education questions administered principally in waves 5, 6, or 8 in the W3 source sample and in wave 8 in the W7 source sample. A participant was considered measured only when a same-wave parent-education question and at least one same-wave valid International Standard Classification of Education 1997 (ISCED-97) value were present. A carried-forward harmonized education value alone did not establish the measurement wave.

Low parental education was defined as either observed parent having ISCED-97 level 0, 1, or 2. The reference category required at least one observed parent and no observed parent below upper-secondary education. The health landmark was the regular interview in the first wave in which parental education was validly observed, and follow-up began only in subsequent waves. This secondary analysis was not folded back into the primary same-interview two-indicator SHARE score.

### 12. Quality control

All participant-wave risk sets were checked for unique person-wave records, post-landmark ordering, positive and strictly increasing times, evaluable events, mutual exclusivity of outcome and death, absence of records after a terminal event, and constancy of baseline exposure. The final quality gate also checked the raw SHARE state correction, sample-flow reconciliation, model uniqueness, finite and ordered confidence intervals, survey and IPCW diagnostics, competing-risk estimates, alternate administrative handling, and required logs.

## Supplementary Results

### 13. Detailed participant flow

**Supplementary Table S5. Cohort-level health-landmark selection**

| **Cohort/source** | **Initial source roster or childhood interview** | **Health-landmark respondent** | **Age ≥50 years** | **Complete primary childhood exposure** |
| --- | --- | --- | --- | --- |
| ELSA-W3 | 19,802 raw harmonized participants | 7,830 wave-3 main-interview respondents after 7,850 life-history interviews | 7,512 | 6,041 |
| SHARE-W3 | 27,949 selected W3 SHARELIFE interviews | 18,890 wave-4 respondents | 18,742 | 18,546 |
| SHARE-W7 | 63,226 selected W7-only SHARELIFE interviews | 32,844 wave-8 respondents | 32,659 | 32,204 |

For ELSA, 6,041 was 80.42% of the 7,512 age-eligible wave-3 life-history/main-interview landmark participants. Of the remaining 1,471, 1,202 had structural item unavailability and 269 had ordinary item-level incompleteness; among the 6,310 respondents for whom all three indicators were in scope, completeness was 95.74%. The smaller percentage obtained by dividing 6,041 by an undifferentiated roster mixes structural non-administration with item-level exposure completeness and is not used as the exposure-completeness percentage.

*Provenance: `03_results/sample_flow_detailed.csv`.*

**Supplementary Table S6. Outcome-specific risk-set flow**

| **Cohort/source** | **Outcome** | **Baseline outcome observed** | **Outcome-free at baseline** | **Broad cause-specific risk-set participant** | **At least one observed outcome follow-up** | **No observed outcome follow-up** | **Known competing death before outcome** | **Administrative censor at any follow-up** |
| --- | --- | --- | --- | --- | --- | --- | --- | --- |
| ELSA-W3 | ADL-6 | 6,041 | 5,002 | 4,801 | 4,688 | 314 | 281 | 0 |
| ELSA-W3 | MM-7 | 6,041 | 4,031 | 3,871 | 3,819 | 212 | 182 | 0 |
| SHARE-W3 | ADL-6 | 18,506 | 16,232 | 14,838 | 14,144 | 2,088 | 2,280 | 2,889 |
| SHARE-W3 | MM-7 | 18,514 | 10,664 | 9,724 | 9,342 | 1,322 | 1,069 | 1,721 |
| SHARE-W7 | ADL-6 | 32,125 | 28,250 | 23,257 | 22,266 | 5,984 | 991 | 1,854 |
| SHARE-W7 | MM-7 | 32,166 | 15,514 | 12,771 | 12,374 | 3,140 | 397 | 1,014 |

The “broad cause-specific risk-set” contains participants with at least one observed assessment or a known competing death. The descriptive death and administrative-censor columns can include participants who had an earlier observed event-free assessment and therefore are not mutually exclusive with “at least one observed outcome follow-up.” Product-weight model sample sizes are smaller because they additionally require a positive baseline survey weight and the monotone first-gap calendar. For the same reason, the broad wave-status event counts in Supplementary Table S9 are not expected to equal the final product-weight model counts in Table 2.

*Provenance: `03_results/sample_flow_detailed.csv`.*

**Supplementary Table S7. Mutually exclusive disposition before any observed post-landmark outcome assessment**

| **Cohort/source** | **Outcome-free N** | **At least one observed assessment, n (%)** | **Death before any observed assessment, n (%)** | **Administrative censor before any observed assessment, n (%)** | **Ordinary missing/nonresponse with no observed assessment, n (%)** |
| --- | --- | --- | --- | --- | --- |
| ELSA-W3, ADL-6 | 5,002 | 4,688 (93.72) | 113 (2.26) | 0 (0.00) | 201 (4.02) |
| ELSA-W3, MM-7 | 4,031 | 3,819 (94.74) | 52 (1.29) | 0 (0.00) | 160 (3.97) |
| SHARE-W3, ADL-6 | 16,232 | 14,144 (87.14) | 694 (4.28) | 1,287 (7.93) | 107 (0.66) |
| SHARE-W3, MM-7 | 10,664 | 9,342 (87.60) | 382 (3.58) | 858 (8.05) | 82 (0.77) |
| SHARE-W7, ADL-6 | 28,250 | 22,266 (78.82) | 991 (3.51) | 1,854 (6.56) | 3,139 (11.11) |
| SHARE-W7, MM-7 | 15,514 | 12,374 (79.76) | 397 (2.56) | 1,014 (6.54) | 1,729 (11.14) |

*Provenance: `03_results/no_followup_reasons.csv`. Percentages use the outcome-free denominator in the frozen file.*

### 14. Follow-up duration and wave-specific observation

**Supplementary Table S8. Follow-up duration in the positive-baseline-weight monotone analysis**

| **Cohort/source** | **Outcome** | **Window** | **N** | **Mean (SD), years** | **Median (IQR), years** | **Range, years** |
| --- | --- | --- | --- | --- | --- | --- |
| ELSA | ADL-6 | waves 4–6 | 4,290 | 5.10 (1.51) | 6.00 (4.00–6.00) | 2.00–6.00 |
| ELSA | ADL-6 | waves 4–9 | 4,290 | 8.13 (3.92) | 8.00 (4.00–12.00) | 2.00–12.00 |
| ELSA | MM-7 | waves 4–6 | 3,424 | 5.15 (1.47) | 6.00 (4.00–6.00) | 2.00–6.00 |
| ELSA | MM-7 | waves 4–9 | 3,424 | 8.10 (3.82) | 8.00 (4.00–12.00) | 2.00–12.00 |
| SHARE-W3 | ADL-6 | available follow-up | 13,863 | 6.55 (3.43) | 6.08 (3.58–10.50) | 0.08–11.42 |
| SHARE-W7 | ADL-6 | wave 9 | 23,026 | 2.10 (0.30) | 2.08 (1.92–2.25) | 0.08–2.92 |
| SHARE-W3 | MM-7 | available follow-up | 9,064 | 5.85 (3.37) | 5.83 (2.25–8.83) | 0.08–11.42 |
| SHARE-W7 | MM-7 | wave 9 | 12,560 | 2.10 (0.29) | 2.08 (1.92–2.25) | 0.08–2.92 |

Follow-up was measured from the outcome-free health landmark to the last observed outcome assessment or known competing death retained by the monotone first-gap analysis. Very short follow-up values reflect exact post-landmark death or interview timing.

*Provenance: `03_results/followup_summary.csv`, generated from the frozen monotone-analysis risk sets.*

**Supplementary Table S9. Scheduled-wave outcome observation and follow-up states**

| **Cohort/source** | **Outcome** | **Wave** | **At risk** | **Observed assessment** | **Incident outcome** | **Known death** | **Administrative censor** | **Structural nonfieldwork** | **Ordinary missing/nonresponse** | **Observed (%)** |
| --- | --- | --- | --- | --- | --- | --- | --- | --- | --- | --- |
| ELSA-W3 | ADL-6 | 4 | 5,002 | 4,515 | 409 | 76 | 0 | 0 | 411 | 90.3 |
| ELSA-W3 | ADL-6 | 5 | 4,517 | 3,909 | 309 | 119 | 0 | 0 | 489 | 86.5 |
| ELSA-W3 | ADL-6 | 6 | 4,089 | 3,408 | 212 | 86 | 0 | 0 | 595 | 83.3 |
| ELSA-W3 | ADL-6 | 7 | 3,791 | 2,925 | 176 | 0 | 0 | 0 | 866 | 77.2 |
| ELSA-W3 | ADL-6 | 8 | 3,615 | 2,506 | 152 | 0 | 0 | 0 | 1,109 | 69.3 |
| ELSA-W3 | ADL-6 | 9 | 3,463 | 2,183 | 146 | 0 | 0 | 0 | 1,280 | 63.0 |
| ELSA-W3 | MM-7 | 4 | 4,031 | 3,677 | 328 | 35 | 0 | 0 | 319 | 91.2 |
| ELSA-W3 | MM-7 | 5 | 3,668 | 3,194 | 283 | 89 | 0 | 0 | 385 | 87.1 |
| ELSA-W3 | MM-7 | 6 | 3,296 | 2,795 | 224 | 58 | 0 | 0 | 443 | 84.8 |
| ELSA-W3 | MM-7 | 7 | 3,014 | 2,352 | 199 | 0 | 0 | 0 | 662 | 78.0 |
| ELSA-W3 | MM-7 | 8 | 2,815 | 1,976 | 211 | 0 | 0 | 0 | 839 | 70.2 |
| ELSA-W3 | MM-7 | 9 | 2,604 | 1,650 | 179 | 0 | 0 | 0 | 954 | 63.4 |
| SHARE-W3 | ADL-6 | 5 | 16,232 | 12,383 | 891 | 501 | 246 | 1,239 | 1,863 | 76.3 |
| SHARE-W3 | ADL-6 | 6 | 14,594 | 10,414 | 736 | 390 | 180 | 1,471 | 2,139 | 71.4 |
| SHARE-W3 | ADL-6 | 7 | 13,288 | 8,860 | 609 | 461 | 354 | 1,448 | 2,165 | 66.7 |
| SHARE-W3 | ADL-6 | 8 | 11,864 | 6,133 | 453 | 478 | 853 | 0 | 4,400 | 51.7 |
| SHARE-W3 | ADL-6 | 9 | 10,080 | 6,378 | 431 | 450 | 1,256 | 0 | 1,996 | 63.3 |
| SHARE-W3 | MM-7 | 5 | 10,664 | 8,235 | 1,263 | 270 | 170 | 752 | 1,237 | 77.2 |
| SHARE-W3 | MM-7 | 6 | 8,961 | 6,288 | 953 | 204 | 126 | 986 | 1,357 | 70.2 |
| SHARE-W3 | MM-7 | 7 | 7,678 | 4,942 | 753 | 209 | 226 | 973 | 1,328 | 64.4 |
| SHARE-W3 | MM-7 | 8 | 6,490 | 3,319 | 507 | 188 | 533 | 0 | 2,450 | 51.1 |
| SHARE-W3 | MM-7 | 9 | 5,262 | 3,266 | 551 | 198 | 666 | 0 | 1,132 | 62.1 |
| SHARE-W7 | ADL-6 | 9 | 28,250 | 22,266 | 1,434 | 991 | 1,854 | 0 | 3,139 | 78.8 |
| SHARE-W7 | MM-7 | 9 | 15,514 | 12,374 | 1,719 | 397 | 1,014 | 0 | 1,729 | 79.8 |

An incident outcome is included in the observed-assessment count. These are broad cause-specific risk-set wave-status counts; Table 2 uses the smaller positive-baseline-weight, monotone first-gap product-weight model set. ELSA waves 7–9 show zero known deaths because the locally available mortality information ended at wave 6; this is the reason the extended ELSA window is explicitly labelled mortality-incomplete.

*Provenance: `03_results/wave_followup_status.csv`.*

### 15. Baseline missingness and selection

**Supplementary Table S10A. Primary-model completeness and multiple-imputation decision**

| **Cohort/source** | **Outcome** | **Target N** | **Primary covariates complete, n (%)** | **Optional covariates complete, n (%)** | **Positive baseline weight, n (%)** | **Education missing, n (%)** | **Partnership missing, n (%)** |
| --- | --- | --- | --- | --- | --- | --- | --- |
| ELSA | ADL-6 | 5,002 | 5,002 (100.0) | 4,619 (92.3) | 4,661 (93.2) | 383 (7.7) | 0 (0.0) |
| ELSA | MM-7 | 4,031 | 4,031 (100.0) | 3,735 (92.7) | 3,707 (92.0) | 296 (7.3) | 0 (0.0) |
| SHARE-W3 | ADL-6 | 16,232 | 16,232 (100.0) | 16,195 (99.8) | 16,091 (99.1) | 0 (0.0) | 34 (0.2) |
| SHARE-W3 | MM-7 | 10,664 | 10,664 (100.0) | 10,638 (99.8) | 10,536 (98.8) | 0 (0.0) | 22 (0.2) |
| SHARE-W7 | ADL-6 | 28,250 | 28,250 (100.0) | 28,230 (99.9) | 27,962 (99.0) | 0 (0.0) | 6 (<0.1) |
| SHARE-W7 | MM-7 | 15,514 | 15,514 (100.0) | 15,500 (99.9) | 15,254 (98.3) | 0 (0.0) | 3 (<0.1) |

Primary covariates were baseline age, sex, and country in SHARE. Optional covariates were education, partnership, and the other baseline health domain. Because every participant in the target risk sets had complete primary covariates, no primary multiple imputation was undertaken. Structural absence of childhood information was not treated as item missingness and was not imputed.

*Provenance: `03_results/baseline_analysis_completeness.csv` and `03_results/baseline_covariate_mi_decision.csv`.*

In ELSA, participants missing adult education were older and more often women. For ADL-6, mean age was 67.3 versus 64.3 years and 72.3% versus 53.0% were women among those with versus without missing education. For MM-7, the corresponding figures were 65.8 versus 63.1 years and 70.6% versus 52.9%. These differences support treating education-adjusted complete-case analyses cautiously rather than replacing the primary age/sex-adjusted model.

**Supplementary Table S10B. ELSA exposure-complete versus exposure-incomplete age-eligible wave-3 landmark respondents**

| **Characteristic** | **Exposure complete** | **Exposure incomplete** | **Signed standardized difference** |
| --- | --- | --- | --- |
| Participants, N | 6,041 | 1,471 | — |
| Age, mean (SD), years | 65.34 (10.00) | 64.44 (11.04) | 0.085 |
| Women, % | 55.3 | 55.9 | −0.012 |
| Married/partnered, % | 72.5 | 59.1 | 0.286 |
| Education: low/primary or less, % of nonmissing | 34.2 | 38.4 | −0.088 |
| Education: middle/secondary, % of nonmissing | 48.2 | 47.5 | 0.013 |
| Education: high/tertiary, % of nonmissing | 17.6 | 14.1 | 0.098 |
| Education missing, % | 8.1 | 7.6 | 0.017 |
| Baseline ADL-6 disability, % | 17.2 | 22.2 | −0.125 |
| Baseline MM-7 multimorbidity, % | 33.3 | 34.5 | −0.025 |

The 1,471 exposure-incomplete respondents comprise 1,202 with structural item unavailability and 269 with ordinary item-level missingness. Signed standardized differences are oriented as exposure complete minus exposure incomplete; their absolute values should be interpreted descriptively rather than as hypothesis tests. Partnership status showed the largest displayed imbalance. Education-category percentages use 5,554 and 1,359 nonmissing observations, respectively.

*Provenance: `03_results/elsa_exposure_missingness_diagnostics.csv` and `03_results/selection_standardized_differences.csv`, contrast `exposure_complete_vs_incomplete`.*

**Supplementary Table S11. Largest non-mechanical standardized difference in selected inclusion comparisons**

| **Cohort/source** | **Outcome** | **Comparison** | **Included/excluded N** | **Variable with largest displayed imbalance** | **Included value** | **Excluded value** | **Signed standardized difference** |
| --- | --- | --- | --- | --- | --- | --- | --- |
| ELSA-W3 | ADL-6 | Broad cause-specific participant vs all later-stage landmark exclusions | 4,801/2,711 | Baseline MM-7 positive | 27.4% | 44.3% | −0.359 |
| ELSA-W3 | ADL-6 | Any observed follow-up vs none | 4,688/314 | Baseline MM-7 positive | 26.5% | 41.4% | −0.319 |
| ELSA-W3 | MM-7 | Broad cause-specific participant vs all later-stage landmark exclusions | 3,871/3,641 | Baseline ADL-6 positive | 9.9% | 26.9% | −0.450 |
| ELSA-W3 | MM-7 | Any observed follow-up vs none | 3,819/212 | Education category 1 | 28.0% | 37.4% | −0.200 |
| SHARE-W3 | ADL-6 | Broad cause-specific participant vs all later-stage landmark exclusions | 14,838/3,904 | Age, years | 67.3 | 71.4 | −0.422 |
| SHARE-W3 | ADL-6 | Any observed follow-up vs none | 14,144/2,088 | Germany | 6.2% | 16.8% | −0.335 |
| SHARE-W3 | MM-7 | Broad cause-specific participant vs all later-stage landmark exclusions | 9,724/9,018 | Age, years | 65.8 | 70.7 | −0.542 |
| SHARE-W3 | MM-7 | Any observed follow-up vs none | 9,342/1,322 | Germany | 6.4% | 17.6% | −0.349 |
| SHARE-W7 | ADL-6 | Broad cause-specific participant vs all later-stage landmark exclusions | 23,257/9,402 | Age, years | 68.5 | 72.2 | −0.391 |
| SHARE-W7 | ADL-6 | Any observed follow-up vs none | 22,266/5,984 | Spain | 1.6% | 12.2% | −0.430 |
| SHARE-W7 | MM-7 | Broad cause-specific participant vs all later-stage landmark exclusions | 12,771/19,888 | Age, years | 66.0 | 71.8 | −0.649 |
| SHARE-W7 | MM-7 | Any observed follow-up vs none | 12,374/3,140 | Spain | 1.1% | 11.9% | −0.448 |

These rows are descriptive selection diagnostics, not evidence that adjustment for the displayed variable alone removes selection bias. The complete standardized-difference output contains all evaluated variables and contrasts.

*Provenance: `03_results/selection_bias_highlights_nonmechanical.csv`; complete underlying contrasts are in `03_results/selection_standardized_differences.csv`.*

### 16. Survey-design and product-weight diagnostics

**Supplementary Table S12. Declared survey design and combined-weight effective sample size**

| **Analysis** | **N before baseline weight** | **N analysed** | **Intervals** | **Events/deaths** | **Strata/PSUs** | **Design df** | **Baseline-weight ESS** | **Participants assigned a pseudo-PSU, N** | **First-gap censored** | **Combined-weight interval ESS** | **Final-person ESS, n (%)** |
| --- | --- | --- | --- | --- | --- | --- | --- | --- | --- | --- | --- |
| ELSA ADL-6, waves 4–9 | 4,591 | 4,290 | 17,435 | 1,252/215 | 10/3,320 | 3,310 | 4,010.1 | 0 | 1,639 | 16,106.0 | 3,943.9 (91.9) |
| ELSA ADL-6, waves 4–6 | 4,591 | 4,290 | 10,936 | 852/215 | 10/3,320 | 3,310 | 4,010.1 | 0 | 860 | 10,152.4 | 3,979.0 (92.8) |
| ELSA MM-7, waves 4–9 | 3,712 | 3,424 | 13,861 | 1,235/145 | 10/2,788 | 2,778 | 3,203.7 | 0 | 1,240 | 12,826.3 | 3,149.3 (92.0) |
| ELSA MM-7, waves 4–6 | 3,712 | 3,424 | 8,812 | 736/145 | 10/2,788 | 2,778 | 3,203.7 | 0 | 654 | 8,193.9 | 3,180.6 (92.9) |
| SHARE-W3 ADL-6, waves 5–9 | 13,977 | 13,863 | 41,635 | 2,721/1,735 | 151/5,306 | 5,155 | 5,590.5 | 4,318 | 7,780 | 16,606.5 | 5,627.3 (40.6) |
| SHARE-W7 ADL-6, wave 9 | 23,257 | 23,026 | 23,026 | 1,432/990 | 566/10,546 | 9,980 | 5,717.2 | 7,227 | 4,993 | 5,730.9 | 5,730.9 (24.9) |
| SHARE-W3 MM-7, waves 5–9 | 9,167 | 9,064 | 24,253 | 3,536/802 | 150/3,892 | 3,742 | 3,391.2 | 2,964 | 4,538 | 8,990.0 | 3,399.9 (37.5) |
| SHARE-W7 MM-7, wave 9 | 12,771 | 12,560 | 12,560 | 1,705/396 | 529/6,603 | 6,074 | 3,060.3 | 3,824 | 2,743 | 3,067.1 | 3,067.1 (24.4) |

Effective sample size (ESS) denotes Kish effective sample size. The large number of SHARE pseudo-primary-sampling-unit participants reflects absent official PSU codes in the local files, not a claim that each participant was an original sampling unit. Participants assigned to a pseudo-stratum because the available stratum code was missing numbered 2 in each ELSA analysis, 3,771 in SHARE-W3 ADL-6, 4,253 in SHARE-W7 ADL-6, 2,607 in SHARE-W3 MM-7, and 2,399 in SHARE-W7 MM-7. The displayed stratum counts include these declared pseudo-strata. The low SHARE effective-sample-size percentages reflect variation in the calibrated baseline weights and should be considered when interpreting precision. Baseline-weight, IPCW, and combined-weight minima, quantiles, maxima, and design details are retained in the source file.

*Provenance: `03_results/survey_ipcw_combined_weight_design_diagnostics.csv`.*

**Supplementary Table S13. Monotone IPCW response-model and weight diagnostics**

| **Cohort/source** | **Outcome/window** | **Response rows** | **Observed/R=0 first gaps** | **Response rate** | **Brier score, numerator/denominator** | **Raw denominator predicted-probability range (before bounding)** | **Raw IPCW p01/median/p99/max** | **Truncated IPCW p01/median/p99/max** |
| --- | --- | --- | --- | --- | --- | --- | --- | --- |
| ELSA | ADL-6; waves 4–9 | 20,157 | 18,518/1,639 | 91.9% | 0.074/0.074 | 0.689–0.973 | 0.878/0.994/1.174/1.382 | 0.878/0.994/1.174/1.174 |
| ELSA | ADL-6; waves 4–6 | 12,363 | 11,503/860 | 93.0% | 0.064/0.064 | 0.791–0.977 | 0.931/0.994/1.099/1.194 | 0.934/0.994/1.099/1.099 |
| ELSA | MM-7; waves 4–9 | 16,124 | 14,884/1,240 | 92.3% | 0.070/0.070 | 0.624–0.971 | 0.897/0.993/1.167/1.307 | 0.900/0.993/1.167/1.170 |
| ELSA | MM-7; waves 4–6 | 10,044 | 9,390/654 | 93.5% | 0.061/0.060 | 0.820–0.973 | 0.952/0.996/1.079/1.126 | 0.952/0.996/1.079/1.083 |
| SHARE-W3 | ADL-6; waves 5–9 | 48,010 | 40,230/7,780 | 83.8% | 0.127/0.127 | 0.407–0.952 | 0.893/1.004/1.091/2.113 | 0.894/1.004/1.091/1.091 |
| SHARE-W7 | ADL-6; wave 9 | 27,259 | 22,266/4,993 | 81.7% | 0.133/0.132 | 0.129–1.000 | 0.951/1.000/1.049/1.156 | 0.951/1.000/1.049/1.049 |
| SHARE-W3 | MM-7; waves 5–9 | 28,283 | 23,745/4,538 | 84.0% | 0.127/0.127 | 0.414–0.957 | 0.882/1.004/1.107/3.364 | 0.883/1.004/1.107/1.107 |
| SHARE-W7 | MM-7; wave 9 | 15,117 | 12,374/2,743 | 81.9% | 0.131/0.130 | 0.059–1.000 | 0.954/1.000/1.061/1.457 | 0.954/1.000/1.061/1.061 |

Both numerator and denominator response models converged in every analysis. No ELSA or SHARE-W3 probability was capped. Six denominator probabilities in SHARE-W7 ADL-6 and three in SHARE-W7 MM-7 were bounded by the declared 0.99 probability ceiling. In the primary SHARE response calendars, administrative censoring contributed 631 W3 ADL-6, 368 W3 MM-7, 1,854 W7 ADL-6, and 1,014 W7 MM-7 terminal R=0 records; the remaining first gaps were ordinary nonresponse or unclassifiable outcome records.

*Provenance: `03_results/survey_ipcw_combined_response_model_diagnostics.csv` and `03_results/survey_ipcw_combined_weight_design_diagnostics.csv`.*

### 17. Full estimator comparison

Supplementary Table S14 compares the highest cohort-specific exposure category with score 0 under all six retained estimators. The table is intentionally redundant: it shows which changes arise from restricting to a monotone response history, applying attrition weights, applying baseline survey weights, or using the declared complex-survey variance. Point estimates from the cluster-robust and full-design versions of the same product-weight model are identical; their confidence intervals differ because the variance calculations differ.

**Supplementary Table S14A. Full estimator comparison for ELSA**

| **Analysis** | **Estimator** | **N** | **Events** | **Deaths** | **HR (95% CI)** |
| --- | --- | --- | --- | --- | --- |
| ADL-6, waves 4–9 | Unweighted cluster-robust available follow-up | 4,801 | 1,404 | 281 | 1.544 (1.280–1.861) |
| ADL-6, waves 4–9 | Baseline survey-weighted available follow-up | 4,485 | 1,336 | 275 | 1.492 (1.212–1.837) |
| ADL-6, waves 4–9 | Monotone first-gap, unweighted | 4,591 | 1,315 | 221 | 1.527 (1.258–1.852) |
| ADL-6, waves 4–9 | Monotone first-gap plus IPCW | 4,591 | 1,315 | 221 | 1.516 (1.249–1.840) |
| ADL-6, waves 4–9 | Baseline weight × IPCW, cluster robust | 4,290 | 1,252 | 215 | 1.470 (1.187–1.820) |
| ADL-6, waves 4–9 | Declared available survey design × baseline weight × IPCW | 4,290 | 1,252 | 215 | 1.470 (1.186–1.822) |
| MM-7, waves 4–9 | Unweighted cluster-robust available follow-up | 3,871 | 1,424 | 182 | 1.225 (0.988–1.519) |
| MM-7, waves 4–9 | Baseline survey-weighted available follow-up | 3,569 | 1,326 | 176 | 1.203 (0.955–1.515) |
| MM-7, waves 4–9 | Monotone first-gap, unweighted | 3,712 | 1,325 | 151 | 1.263 (1.012–1.575) |
| MM-7, waves 4–9 | Monotone first-gap plus IPCW | 3,712 | 1,325 | 151 | 1.261 (1.010–1.574) |
| MM-7, waves 4–9 | Baseline weight × IPCW, cluster robust | 3,424 | 1,235 | 145 | 1.240 (0.978–1.573) |
| MM-7, waves 4–9 | Declared available survey design × baseline weight × IPCW | 3,424 | 1,235 | 145 | 1.240 (0.979–1.571) |
| ADL-6, waves 4–6 | Unweighted cluster-robust available follow-up | 4,797 | 930 | 281 | 1.513 (1.200–1.908) |
| ADL-6, waves 4–6 | Baseline survey-weighted available follow-up | 4,482 | 891 | 275 | 1.456 (1.124–1.886) |
| ADL-6, waves 4–6 | Monotone first-gap, unweighted | 4,591 | 889 | 221 | 1.487 (1.171–1.888) |
| ADL-6, waves 4–6 | Monotone first-gap plus IPCW | 4,591 | 889 | 221 | 1.482 (1.167–1.882) |
| ADL-6, waves 4–6 | Baseline weight × IPCW, cluster robust | 4,290 | 852 | 215 | 1.428 (1.096–1.862) |
| ADL-6, waves 4–6 | Declared available survey design × baseline weight × IPCW | 4,290 | 852 | 215 | 1.428 (1.092–1.868) |
| MM-7, waves 4–6 | Unweighted cluster-robust available follow-up | 3,867 | 835 | 182 | 1.365 (1.055–1.767) |
| MM-7, waves 4–6 | Baseline survey-weighted available follow-up | 3,566 | 773 | 176 | 1.346 (1.021–1.775) |
| MM-7, waves 4–6 | Monotone first-gap, unweighted | 3,712 | 795 | 151 | 1.383 (1.062–1.801) |
| MM-7, waves 4–6 | Monotone first-gap plus IPCW | 3,712 | 795 | 151 | 1.388 (1.066–1.807) |
| MM-7, waves 4–6 | Baseline weight × IPCW, cluster robust | 3,424 | 736 | 145 | 1.355 (1.018–1.803) |
| MM-7, waves 4–6 | Declared available survey design × baseline weight × IPCW | 3,424 | 736 | 145 | 1.355 (1.021–1.796) |

The waves 4–9 ELSA mortality file contains no deaths after wave 6. Its outcome hazard is therefore reported as an extended mortality-incomplete sensitivity, not as a fully observed competing-risk window.

**Supplementary Table S14B. Full estimator comparison for SHARE**

| **Analysis** | **Estimator** | **N** | **Events** | **Deaths** | **HR (95% CI)** |
| --- | --- | --- | --- | --- | --- |
| W3 ADL-6, waves 5–9 | Unweighted cluster-robust available follow-up | 14,838 | 3,120 | 2,280 | 1.188 (1.037–1.361) |
| W3 ADL-6, waves 5–9 | Baseline survey-weighted available follow-up | 14,715 | 3,111 | 2,277 | 1.290 (1.031–1.614) |
| W3 ADL-6, waves 5–9 | Monotone first-gap, unweighted | 13,977 | 2,729 | 1,736 | 1.239 (1.069–1.436) |
| W3 ADL-6, waves 5–9 | Monotone first-gap plus IPCW | 13,977 | 2,729 | 1,736 | 1.236 (1.066–1.432) |
| W3 ADL-6, waves 5–9 | Baseline weight × IPCW, cluster robust | 13,863 | 2,721 | 1,735 | 1.359 (1.066–1.733) |
| W3 ADL-6, waves 5–9 | Declared available survey design × baseline weight × IPCW | 13,863 | 2,721 | 1,735 | 1.359 (1.077–1.715) |
| W3 MM-7, waves 5–9 | Unweighted cluster-robust available follow-up | 9,724 | 4,027 | 1,069 | 1.214 (1.091–1.351) |
| W3 MM-7, waves 5–9 | Baseline survey-weighted available follow-up | 9,613 | 4,004 | 1,066 | 1.339 (1.103–1.626) |
| W3 MM-7, waves 5–9 | Monotone first-gap, unweighted | 9,167 | 3,554 | 803 | 1.238 (1.104–1.388) |
| W3 MM-7, waves 5–9 | Monotone first-gap plus IPCW | 9,167 | 3,554 | 803 | 1.236 (1.102–1.385) |
| W3 MM-7, waves 5–9 | Baseline weight × IPCW, cluster robust | 9,064 | 3,536 | 802 | 1.340 (1.104–1.628) |
| W3 MM-7, waves 5–9 | Declared available survey design × baseline weight × IPCW | 9,064 | 3,536 | 802 | 1.340 (1.092–1.645) |
| W7 ADL-6, wave 9 | Unweighted cluster-robust available follow-up | 23,257 | 1,434 | 991 | 1.468 (1.189–1.814) |
| W7 ADL-6, wave 9 | Baseline survey-weighted available follow-up | 23,026 | 1,432 | 990 | 1.607 (1.061–2.434) |
| W7 ADL-6, wave 9 | Monotone first-gap, unweighted | 23,257 | 1,434 | 991 | 1.468 (1.189–1.814) |
| W7 ADL-6, wave 9 | Monotone first-gap plus IPCW | 23,257 | 1,434 | 991 | 1.463 (1.185–1.808) |
| W7 ADL-6, wave 9 | Baseline weight × IPCW, cluster robust | 23,026 | 1,432 | 990 | 1.602 (1.047–2.451) |
| W7 ADL-6, wave 9 | Declared available survey design × baseline weight × IPCW | 23,026 | 1,432 | 990 | 1.602 (1.058–2.427) |
| W7 MM-7, wave 9 | Unweighted cluster-robust available follow-up | 12,771 | 1,719 | 397 | 1.047 (0.889–1.233) |
| W7 MM-7, wave 9 | Baseline survey-weighted available follow-up | 12,560 | 1,705 | 396 | 0.929 (0.679–1.270) |
| W7 MM-7, wave 9 | Monotone first-gap, unweighted | 12,771 | 1,719 | 397 | 1.047 (0.889–1.233) |
| W7 MM-7, wave 9 | Monotone first-gap plus IPCW | 12,771 | 1,719 | 397 | 1.046 (0.889–1.232) |
| W7 MM-7, wave 9 | Baseline weight × IPCW, cluster robust | 12,560 | 1,705 | 396 | 0.928 (0.682–1.262) |
| W7 MM-7, wave 9 | Declared available survey design × baseline weight × IPCW | 12,560 | 1,705 | 396 | 0.928 (0.678–1.269) |

The SHARE-W7 available-follow-up and monotone first-gap samples coincide because only wave 9 follows the wave-8 health landmark. The slight difference between the unweighted and IPCW point estimates arises from response weighting at that single scheduled assessment. Figure S1 provides a compact visual comparison of the five distinct estimator displays while retaining the mortality qualification for the extended ELSA window.

*Provenance for Tables S14A–B: `03_results/key_results_comparison.csv`.*

### 18. Category-specific product-weight estimates

**Supplementary Table S15. Product-weight hazard ratios using declared available survey-design information, by exposure category**

| **Analysis** | **Exposure category vs 0** | **N** | **Events/deaths** | **HR (95% CI)** | **P value** |
| --- | --- | --- | --- | --- | --- |
| ELSA ADL-6, waves 4–9 | 1 | 4,290 | 1,252/215 | 1.030 (0.896–1.184) | 0.679 |
| ELSA ADL-6, waves 4–9 | ≥2 | 4,290 | 1,252/215 | 1.470 (1.186–1.822) | <0.001 |
| ELSA ADL-6, waves 4–6 | 1 | 4,290 | 852/215 | 1.053 (0.890–1.246) | 0.547 |
| ELSA ADL-6, waves 4–6 | ≥2 | 4,290 | 852/215 | 1.428 (1.092–1.868) | 0.009 |
| ELSA MM-7, waves 4–9 | 1 | 3,424 | 1,235/145 | 1.028 (0.891–1.186) | 0.704 |
| ELSA MM-7, waves 4–9 | ≥2 | 3,424 | 1,235/145 | 1.240 (0.979–1.571) | 0.075 |
| ELSA MM-7, waves 4–6 | 1 | 3,424 | 736/145 | 0.997 (0.827–1.202) | 0.972 |
| ELSA MM-7, waves 4–6 | ≥2 | 3,424 | 736/145 | 1.355 (1.021–1.796) | 0.035 |
| SHARE-W3 ADL-6, waves 5–9 | 1 | 13,863 | 2,721/1,735 | 1.091 (0.861–1.382) | 0.471 |
| SHARE-W3 ADL-6, waves 5–9 | 2 | 13,863 | 2,721/1,735 | 1.359 (1.077–1.715) | 0.010 |
| SHARE-W7 ADL-6, wave 9 | 1 | 23,026 | 1,432/990 | 1.357 (0.880–2.092) | 0.167 |
| SHARE-W7 ADL-6, wave 9 | 2 | 23,026 | 1,432/990 | 1.602 (1.058–2.427) | 0.026 |
| SHARE-W3 MM-7, waves 5–9 | 1 | 9,064 | 3,536/802 | 1.260 (1.030–1.542) | 0.024 |
| SHARE-W3 MM-7, waves 5–9 | 2 | 9,064 | 3,536/802 | 1.340 (1.092–1.645) | 0.005 |
| SHARE-W7 MM-7, wave 9 | 1 | 12,560 | 1,705/396 | 0.842 (0.616–1.150) | 0.279 |
| SHARE-W7 MM-7, wave 9 | 2 | 12,560 | 1,705/396 | 0.928 (0.678–1.269) | 0.639 |

These are cohort-specific contrasts. The category labels do not imply that ELSA ≥2 and SHARE 2 represent the same latent exposure level.

*Provenance: `03_results/survey_ipcw_combined_results.csv`.*

### 19. Weighted competing-risk absolute risks

**Supplementary Table S16. Standardized outcome risk for the highest cohort-specific exposure category versus 0**

| **Cohort/source** | **Outcome** | **Horizon** | **Risk, category 0 (95% CI)** | **Risk, highest category (95% CI)** | **Risk difference, percentage points (95% CI)** | **Risk ratio (95% CI)** |
| --- | --- | --- | --- | --- | --- | --- |
| ELSA | ADL-6 | 6 years | 19.77% (18.32–21.24) | 26.87% (21.73–32.37) | +7.10 (+2.02 to +12.89) | 1.359 (1.103–1.671) |
| ELSA | MM-7 | 6 years | 21.58% (19.89–23.36) | 28.18% (22.26–34.78) | +6.61 (+0.08 to +12.88) | 1.306 (1.004–1.621) |
| SHARE-W3 | ADL-6 | 6 years | 14.38% (11.43–17.92) | 18.93% (17.80–20.35) | +4.54 (+0.70 to +8.02) | 1.316 (1.038–1.693) |
| SHARE-W3 | MM-7 | 6 years | 28.57% (24.18–33.56) | 36.56% (34.22–39.01) | +7.99 (+2.40 to +13.09) | 1.280 (1.071–1.521) |
| SHARE-W7 | ADL-6 | 2 years | 4.46% (3.11–6.64) | 7.07% (6.36–8.13) | +2.61 (+0.38 to +4.41) | 1.584 (1.059–2.359) |
| SHARE-W7 | MM-7 | 2 years | 12.25% (9.62–15.80) | 11.25% (9.99–13.00) | −1.01 (−4.75 to +2.30) | 0.918 (0.697–1.243) |

The ELSA and SHARE-W3 estimates use six-year horizons, but SHARE-W3 absolute risks use the common waves 5–7 window rather than all waves 5–9. The SHARE-W7 estimate is necessarily two-year. Confidence intervals come from joint survey-linearized coefficient simulation and should be interpreted with the uncertainty limitations described in Supplementary Methods section 9.

Full category-specific outcome and competing-death risks at all estimable horizons are retained in the machine-readable files.

*Provenance: `03_results/weighted_competing_risk_absolute_risks.csv` and `03_results/weighted_competing_risk_absolute_contrasts.csv`.*

### 20. Restricted follow-up-window sensitivities

**Supplementary Table S17. Common first-follow-up or approximately two-year association window**

| **Cohort/source** | **Outcome** | **Window** | **N, weighted/unweighted** | **Baseline survey-weighted HR (95% CI)** | **Unweighted HR (95% CI)** |
| --- | --- | --- | --- | --- | --- |
| ELSA | ADL-6 | Wave 4, two-year | 4,290/4,591 | 1.949 (1.391–2.729) | 2.034 (1.494–2.769) |
| ELSA | MM-7 | Wave 4, two-year | 3,424/3,712 | 1.413 (0.931–2.143) | 1.434 (0.972–2.116) |
| SHARE-W3 | ADL-6 | First post-landmark assessment | 12,776/12,884 | 1.707 (1.019–2.859) | 1.435 (1.091–1.889) |
| SHARE-W3 | MM-7 | First post-landmark assessment | 8,408/8,505 | 1.351 (0.987–1.850) | 1.152 (0.951–1.397) |
| SHARE-W7 | ADL-6 | Wave 9, first post-landmark assessment | 23,026/23,257 | 1.607 (1.061–2.434) | 1.468 (1.189–1.814) |
| SHARE-W7 | MM-7 | Wave 9, first post-landmark assessment | 12,560/12,771 | 0.929 (0.679–1.270) | 1.047 (0.889–1.233) |

These first-follow-up models use the baseline survey weight alone and do not add a multiwave IPCW sequence. They show that the ADL-6 association was positive in each inception sample at the first post-landmark assessment, whereas the MM-7 association was less consistent.

*Provenance: `03_results/sampling_weighted_vs_unweighted_comparison.csv`.*

**Supplementary Table S18. ELSA locally mortality-ascertained versus extended mortality-incomplete product-weight windows**

| **Outcome** | **Waves 4–6 HR (95% CI)** | **Waves 4–9 HR (95% CI)** | **Interpretation of window comparison** |
| --- | --- | --- | --- |
| ADL-6 | 1.428 (1.092–1.868) | 1.470 (1.186–1.822) | Similar constant-association summaries across the overlapping original wave-3 landmark windows; the lagged analysis in Table S26D attenuated after wave-4 onset was excluded |
| MM-7 | 1.355 (1.021–1.796) | 1.240 (0.979–1.571) | Attenuation and loss of interval exclusion of 1 in the extended window |

Both columns use declared available survey-design information and the baseline survey weight multiplied by truncated stabilized IPCW. The waves 4–9 estimate cannot fully account for competing death after wave 6.

*Provenance: `03_results/survey_ipcw_combined_high_exposure_summary.csv`.*

### 21. Alternative SHARE weight and administrative-censor handling

**Supplementary Table S19. SHARE exposure-wave cross-sectional versus adjacent-wave longitudinal weight candidates**

| **SHARE source** | **Outcome/window** | **Cross-sectional candidate HR (95% CI)** | **Longitudinal candidate HR (95% CI)** | **Confidence-limit crossing of 1 changed** |
| --- | --- | --- | --- | --- |
| W3 | ADL-6, available follow-up | 1.290 (1.031–1.614) | 1.290 (1.032–1.614) | No |
| W3 | ADL-6, first follow-up | 1.707 (1.019–2.859) | 1.735 (1.038–2.899) | No |
| W3 | MM-7, available follow-up | 1.339 (1.103–1.626) | 1.335 (1.098–1.623) | No |
| W3 | MM-7, first follow-up | 1.351 (0.987–1.850) | 1.334 (0.968–1.838) | No |
| W7 | ADL-6, available follow-up | 1.607 (1.061–2.434) | 1.699 (1.075–2.685) | No |
| W7 | ADL-6, first follow-up | 1.607 (1.061–2.434) | 1.699 (1.075–2.685) | No |
| W7 | MM-7, available follow-up | 0.929 (0.679–1.270) | 0.984 (0.717–1.351) | No |
| W7 | MM-7, first follow-up | 0.929 (0.679–1.270) | 0.984 (0.717–1.351) | No |

The cross-sectional candidates were cciw_w3 and cciw_w7_SHL; the adjacent-wave longitudinal candidates were cliw_d and cliw_h. W7 available and first follow-up are the same single wave-9 interval and are shown twice only to preserve the estimator inventory. Official documentation established the general start-wave targets of the weight candidates, but the weights were not recalibrated after exposure-complete, age, positive-weight, and outcome-free restrictions; calibration to each final restricted analytic population therefore remains uncertain.

Table S19 compares baseline survey-weighted available-follow-up models. It is a weight-candidate sensitivity and does not additionally apply the monotone IPCW sequence.

*Provenance: `03_results/share_cross_sectional_vs_longitudinal_weight_hr_comparison.csv` and `00_protocol/share_structural_fieldwork_weight_audit_2026-07-23.md`.*

**Supplementary Table S20. SHARE administrative-censor handling sensitivity**

| **SHARE source** | **Outcome** | **Primary terminal R=0, N; HR (95% CI)** | **Independent administrative truncation, N; HR (95% CI)** | **Log-HR shift** |
| --- | --- | --- | --- | --- |
| W3 | ADL-6 | 13,863; 1.359 (1.077–1.715) | 13,863; 1.356 (1.074–1.711) | −0.002 |
| W3 | MM-7 | 9,064; 1.340 (1.092–1.645) | 9,064; 1.342 (1.093–1.648) | +0.001 |
| W7 | ADL-6 | 23,026; 1.602 (1.058–2.427) | 23,026; 1.601 (1.056–2.428) | −0.001 |
| W7 | MM-7 | 12,560; 0.928 (0.678–1.269) | 12,560; 0.928 (0.679–1.270) | +0.001 |

The two administrative-censor strategies gave nearly identical estimates. This agreement does not validate either censoring assumption; it only shows that the reported exposure coefficients were numerically insensitive to this declared alternative in the available data.

*Provenance: `03_results/survey_ipcw_combined_share_admin_censor_handling_sensitivity.csv`. Corresponding absolute-risk sensitivity outputs are in `weighted_competing_risk_admin_independent_truncation_absolute_risks.csv` and `weighted_competing_risk_admin_independent_truncation_absolute_contrasts.csv`.*

### 22. SHARE parental education at its true measurement landmark

Among 27,949 W3 SHARELIFE participants, 15,273 had a valid later parent-education measurement: 15,220 at wave 5, 49 at wave 6, and 4 at wave 8. Among 63,226 W7-only SHARELIFE participants, 9,198 had a valid wave-8 measurement. Low parental education was present in 13,145 of 15,273 W3-source participants (86.1%) and 7,980 of 9,198 W7-source participants (86.8%).

**Supplementary Table S21. True-landmark parental-education analysis flow**

| **Source** | **Outcome** | **Baseline outcome-free N** | **Any observed follow-up N** | **Model-evaluable N** | **Outcome events** | **Competing deaths** |
| --- | --- | --- | --- | --- | --- | --- |
| W3 | ADL-6 | 13,283 | 11,511 | 12,140 | 2,302 | 1,614 |
| W3 | MM-7 | 7,773 | 6,761 | 7,069 | 2,507 | 666 |
| W7 | ADL-6 | 8,066 | 6,548 | 6,929 | 400 | 381 |
| W7 | MM-7 | 5,073 | 4,144 | 4,344 | 750 | 200 |

The model-evaluable set includes a participant with an observed post-landmark assessment or a known competing death, so it can exceed “any observed follow-up.”

*Provenance: `03_results/share_parent_education_true_landmark_flow.csv`.*

**Supplementary Table S22. Low parental education and incident outcomes at the true measurement landmark**

| **Source** | **Outcome** | **Exposure set** | **Estimator** | **N** | **Events/deaths** | **HR (95% CI)** |
| --- | --- | --- | --- | --- | --- | --- |
| W3 | ADL-6 | At least one parent observed | Unweighted, cluster robust | 12,140 | 2,302/1,614 | 1.122 (0.982–1.283) |
| W3 | ADL-6 | At least one parent observed | Survey weighted | 12,128 | 2,300/1,612 | 1.074 (0.886–1.301) |
| W3 | ADL-6 | Both parents observed | Unweighted, cluster robust | 11,903 | 2,251/1,580 | 1.121 (0.979–1.285) |
| W3 | ADL-6 | Both parents observed | Survey weighted | 11,891 | 2,249/1,578 | 1.083 (0.893–1.312) |
| W7 | ADL-6 | At least one parent observed | Unweighted, cluster robust | 6,929 | 400/381 | 1.170 (0.808–1.694) |
| W7 | ADL-6 | At least one parent observed | Survey weighted | 6,927 | 400/381 | 1.059 (0.627–1.788) |
| W7 | ADL-6 | Both parents observed | Unweighted, cluster robust | 6,762 | 389/374 | 1.137 (0.779–1.660) |
| W7 | ADL-6 | Both parents observed | Survey weighted | 6,760 | 389/374 | 1.054 (0.623–1.784) |
| W3 | MM-7 | At least one parent observed | Unweighted, cluster robust | 7,069 | 2,507/666 | 1.168 (1.039–1.314) |
| W3 | MM-7 | At least one parent observed | Survey weighted | 7,060 | 2,503/665 | 1.090 (0.915–1.299) |
| W3 | MM-7 | Both parents observed | Unweighted, cluster robust | 6,938 | 2,460/647 | 1.160 (1.030–1.307) |
| W3 | MM-7 | Both parents observed | Survey weighted | 6,929 | 2,456/646 | 1.099 (0.925–1.306) |
| W7 | MM-7 | At least one parent observed | Unweighted, cluster robust | 4,344 | 750/200 | 1.396 (1.101–1.769) |
| W7 | MM-7 | At least one parent observed | Survey weighted | 4,342 | 750/200 | 1.092 (0.775–1.540) |
| W7 | MM-7 | Both parents observed | Unweighted, cluster robust | 4,242 | 735/196 | 1.388 (1.092–1.765) |
| W7 | MM-7 | Both parents observed | Survey weighted | 4,240 | 735/196 | 1.086 (0.769–1.533) |

Models adjusted for baseline age, sex, country, flexible interval hazard, and interval duration. Survey-weighted confidence intervals included 1 for both outcomes in both source samples. No IPCW analysis was added for this secondary exposure. These estimates apply to the selected subset with a valid later parental-education measurement and should not be compared directly with the ELSA or two-indicator SHARE score as if the exposures were interchangeable.

*Provenance: `03_results/share_parent_education_true_landmark_results.csv`, with timing and design diagnostics in `share_parent_education_measurement_wave_diagnostics.csv` and `share_parent_education_true_landmark_design_diagnostics.csv`.*

### 23. SHARE selection from exposure interview to health landmark

The post-landmark IPCW begins only after the wave-4 or wave-8 health landmark. Selection between the SHARELIFE exposure interview and that landmark was therefore examined separately. “Reached” required an observed main interview at the next regular health wave; age, exposure completeness, baseline outcome, and outcome-free restrictions were applied only afterwards.

**Supplementary Table S23A. SHARE exposure-interview-to-health-landmark disposition**

| **Source** | **SHARELIFE source, N** | **Reached next-wave health landmark, n (%)** | **Did not reach, N** | **Death/end of life, n (% of not reached)** | **Country/batch not fielded, n (%)** | **Household/person nonparticipation, n (%)** | **Known alive without main interview, n (%)** | **Administrative/other, n (%)** |
| --- | --- | --- | --- | --- | --- | --- | --- | --- |
| SHARE-W3 | 27,949 | 18,890 (67.6) | 9,059 | 1,004 (11.1) | 3,655 (40.3) | 3,913 (43.2) | 392 (4.3) | 95 (1.0) |
| SHARE-W7-only | 63,226 | 32,844 (51.9) | 30,382 | 3,178 (10.5) | 0 (0.0) | 10,333 (34.0) | 14,418 (47.5) | 2,453 (8.1) |

Categories are mutually exclusive and reconcile to each source population. “Administrative/other” combines administrative out-of-sample status with the small vital-status-unknown category.

**Supplementary Table S23B. Health-landmark participation by childhood exposure category**

| **Source** | **Exposure category at SHARELIFE** | **Source N** | **Reached landmark, N** | **Reached (%)** |
| --- | --- | --- | --- | --- |
| SHARE-W3 | Score 0 | 2,917 | 2,215 | 75.9 |
| SHARE-W3 | Score 1 | 7,248 | 5,266 | 72.7 |
| SHARE-W3 | Score 2 | 17,425 | 11,212 | 64.3 |
| SHARE-W3 | Exposure incomplete | 359 | 197 | 54.9 |
| SHARE-W7-only | Score 0 | 7,490 | 4,149 | 55.4 |
| SHARE-W7-only | Score 1 | 18,317 | 9,974 | 54.5 |
| SHARE-W7-only | Score 2 | 36,022 | 18,263 | 50.7 |
| SHARE-W7-only | Exposure incomplete | 1,397 | 458 | 32.8 |

Participants reaching the health landmark were modestly younger than those not reaching it (standardized mean difference [SMD] −0.106 in W3 and −0.047 in W7); sex SMDs were 0.026 and 0.037, and exposure-completeness SMDs were 0.063 and 0.115. Country-specific landmark-participation rates also varied. These diagnostics demonstrate selection but do not identify or remove all bias from survival and participation before the health landmark. Figure S3A visualizes the exposure-category gradient in landmark participation.

*Provenance: `03_results/share_source_to_landmark_overall.csv`, `share_source_to_landmark_reason_counts.csv`, `share_source_to_landmark_by_exposure.csv`, `share_source_to_landmark_standardized_differences.csv`, and `share_source_to_landmark_validation_checks.csv`.*

### 24. SHARE longitudinal-weight full-chain sensitivity

The adjacent-wave longitudinal weights (cliw_d for W3–W4 and cliw_h for W7–W8) were substituted for the exposure-wave cross-sectional candidates while retaining the same frozen risk sets, post-landmark monotone IPCW, outcome/death definitions, covariates, and variance approximation. Thus, only the baseline weight candidate changed.

**Supplementary Table S24A. Cross-sectional versus adjacent-wave longitudinal baseline weights in the full post-landmark IPCW hazard analysis**

| **SHARE source** | **Outcome** | **Cross-sectional candidate HR (95% CI)** | **Longitudinal candidate HR (95% CI)** | **Relative HR change** | **CI classification changed** |
| --- | --- | --- | --- | --- | --- |
| W3 | ADL-6 | 1.359 (1.077–1.715) | 1.349 (1.062–1.713) | −0.7% | No |
| W3 | MM-7 | 1.340 (1.092–1.645) | 1.337 (1.088–1.644) | −0.2% | No |
| W7 | ADL-6 | 1.602 (1.058–2.427) | 1.694 (1.070–2.679) | +5.7% | No |
| W7 | MM-7 | 0.928 (0.678–1.269) | 0.984 (0.716–1.351) | +6.0% | No |

**Supplementary Table S24B. Cross-sectional versus adjacent-wave longitudinal baseline weights in standardized absolute-risk analyses**

| **SHARE source** | **Outcome/horizon** | **Cross-sectional score 0 / highest risk; RD (95% CI), percentage points** | **Longitudinal score 0 / highest risk; RD (95% CI), percentage points** |
| --- | --- | --- | --- |
| W3 | ADL-6/6 years | 14.4% / 18.9%; 4.5 (0.7–8.0) | 14.3% / 18.8%; 4.5 (0.6–7.8) |
| W3 | MM-7/6 years | 28.6% / 36.6%; 8.0 (2.4–13.1) | 28.6% / 37.0%; 8.4 (2.9–13.7) |
| W7 | ADL-6/2 years | 4.5% / 7.1%; 2.6 (0.4–4.4) | 3.7% / 6.1%; 2.5 (0.5–4.1) |
| W7 | MM-7/2 years | 12.3% / 11.2%; −1.0 (−4.7–2.3) | 11.4% / 11.0%; −0.4 (−4.0–2.7) |

The longitudinal-weight analyses apply to source-wave SHARELIFE respondents who survived and responded at the next regular health landmark, met the subsequent study restrictions, and had a positive official longitudinal weight. They do not recover people who died or were absent before the landmark, and post-landmark IPCW remains assumption-dependent. The unchanged inferential pattern supports robustness to the candidate baseline weight without establishing absence of selection bias. Figure S3B places the two weight-candidate estimates side by side.

*Provenance: `03_results/share_longitudinal_weight_full_chain_summary.csv`, `share_cross_vs_longitudinal_full_chain_hazard_comparison.csv`, `share_cross_vs_longitudinal_full_chain_absolute_contrast_comparison.csv`, and `share_longitudinal_weight_full_chain_validation_checks.csv`.*

### 25. ELSA indicator composition and indicator-specific sensitivity

The ELSA score contains two distinct but related health indicators and one uncommon material-hardship indicator. The following analyses were exploratory and did not replace the dated post-review categorical composite contrast.

**Supplementary Table S25A. All combinations of the three ELSA childhood indicators at the wave-3 landmark**

| **Childhood self-rated health indicator** | **Health-related school absence** | **Childhood financial hardship** | **Score** | **N (%)** |
| --- | --- | --- | --- | --- |
| 0 | 0 | 0 | 0 | 4,333 (71.73) |
| 0 | 0 | 1 | 1 | 96 (1.59) |
| 0 | 1 | 0 | 1 | 892 (14.77) |
| 0 | 1 | 1 | 2 | 18 (0.30) |
| 1 | 0 | 0 | 1 | 281 (4.65) |
| 1 | 0 | 1 | 2 | 13 (0.22) |
| 1 | 1 | 0 | 2 | 392 (6.49) |
| 1 | 1 | 1 | 3 | 16 (0.26) |

Among the 439 participants with a score of at least 2, 408 (92.9%) had both health-related indicators, 392 (89.3%) had both health indicators without financial hardship, and 47 (10.7%) had financial hardship. The phi correlation between the two health-related indicators was 0.319; they were related but not duplicate variables.

**Supplementary Table S25B. Indicator-specific ELSA cause-specific hazard ratios in the mortality-ascertained waves 4–6 window**

| **Indicator** | **ADL-6 separate HR (95% CI)** | **ADL-6 mutually adjusted HR (95% CI)** | **MM-7 separate HR (95% CI)** | **MM-7 mutually adjusted HR (95% CI)** |
| --- | --- | --- | --- | --- |
| Childhood self-rated health fair/poor/varied greatly | 1.29 (1.04–1.61) | 1.26 (1.01–1.57) | 1.31 (1.04–1.64) | 1.30 (1.03–1.65) |
| Missed school ≥1 month because of health | 1.11 (0.94–1.31) | 1.05 (0.89–1.24) | 1.07 (0.90–1.29) | 1.01 (0.84–1.21) |
| Severe financial hardship before age 16 | 1.33 (0.86–2.07) | 1.30 (0.83–2.01) | 1.01 (0.63–1.61) | 0.98 (0.61–1.57) |

Separate models contained one indicator at a time; mutually adjusted models contained all three indicators. Both used baseline age, sex, flexible scheduled-wave hazard, interval-duration offset, and wave-3 baseline weight × stabilized monotone IPCW. Confidence intervals were not adjusted for multiplicity.

**Supplementary Table S25C. Six-year standardized risk differences for separate-indicator ELSA models**

| **Indicator** | **ADL-6 RD (95% CI), percentage points** | **MM-7 RD (95% CI), percentage points** |
| --- | --- | --- |
| Childhood self-rated health fair/poor/varied greatly | 5.1 (1.0–9.7) | 5.5 (0.7–10.9) |
| Missed school ≥1 month because of health | 1.9 (−0.9–5.1) | 1.4 (−2.1–5.2) |
| Severe financial hardship before age 16 | 5.4 (−3.2–15.8) | 0.1 (−7.7–10.9) |

The indicator-specific results most clearly support an association for adverse childhood self-rated health. They do not establish that this indicator is the unique causal driver of the composite association. Financial hardship was uncommon: the final MM-7 model contained 83 exposed participants and 19 exposed events, so its estimate is especially imprecise. Extended waves 4–9 models showed the same broad pattern but remain mortality-incomplete and are retained only in the machine-readable sensitivity output. Figure S2 jointly displays the indicator-specific estimates and the mutually exclusive composition of the ELSA highest-score category.

*Provenance: `00_protocol/SAP_amendment_03_ELSA_indicator_specific_exposure_2026-07-25.md`, `03_results/elsa_component_combination_distribution.csv`, `elsa_component_separate_hazards.csv`, `elsa_component_mutually_adjusted_hazards.csv`, `elsa_component_absolute_contrasts.csv`, and `elsa_component_sensitivity_qa.csv`.*

### 26. Unplanned final statistical-closure diagnostics

These diagnostics used the same product weight, risk set, covariates, interval-duration offset, and declared variance approximation as the Table-2 analysis. They were added during final post-review closure, were not prospectively specified, and did not replace the frozen main model.

**Supplementary Table S26A. Sensitivity of the highest-versus-zero hazard ratio to adding baseline age squared**

| **Analysis** | **Linear-age HR (95% CI)** | **Age + age² HR (95% CI)** | **Relative HR change** | **age² p** |
| --- | --- | --- | --- | --- |
| ELSA ADL-6, waves 4–6 | 1.428 (1.092–1.868) | 1.431 (1.087–1.884) | +0.2% | 0.171 |
| ELSA ADL-6, waves 4–9 | 1.470 (1.186–1.822) | 1.475 (1.184–1.838) | +0.4% | 0.089 |
| ELSA MM-7, waves 4–6 | 1.355 (1.021–1.796) | 1.340 (1.013–1.773) | −1.1% | 0.027 |
| ELSA MM-7, waves 4–9 | 1.240 (0.979–1.571) | 1.230 (0.971–1.557) | −0.8% | 0.014 |
| SHARE-W3 ADL-6 | 1.359 (1.077–1.715) | 1.356 (1.074–1.713) | −0.2% | 0.741 |
| SHARE-W3 MM-7 | 1.340 (1.092–1.645) | 1.323 (1.078–1.622) | −1.3% | 0.015 |
| SHARE-W7 ADL-6 | 1.602 (1.058–2.427) | 1.690 (1.115–2.561) | +5.5% | <0.001 |
| SHARE-W7 MM-7 | 0.928 (0.678–1.269) | 0.909 (0.665–1.244) | −2.0% | 0.024 |

Five age-squared terms had *p*<0.05, indicating that the linear-age form did not fully describe baseline age associations in several models. Nevertheless, the highest-category exposure HR changed by no more than 5.5%, and no confidence-interval classification changed; this diagnostic supports stability of the reported exposure contrast, not validation of age linearity.

**Supplementary Table S26B. Global tests of exposure category × scheduled wave**

| **Analysis** | **Scheduled waves** | **Global Wald F** | **Degrees of freedom** | **p** | **Estimability** |
| --- | --- | --- | --- | --- | --- |
| ELSA ADL-6, waves 4–6 | 4, 5, 6 | 2.943 | 4, 3310 | 0.019 | Estimable |
| ELSA ADL-6, waves 4–9 | 4–9 | 1.536 | 10, 3310 | 0.120 | Estimable |
| ELSA MM-7, waves 4–6 | 4, 5, 6 | 0.664 | 4, 2778 | 0.617 | Estimable |
| ELSA MM-7, waves 4–9 | 4–9 | 0.520 | 10, 2778 | 0.877 | Estimable |
| SHARE-W3 ADL-6 | 5–9 | 0.719 | 8, 5155 | 0.675 | Estimable |
| SHARE-W3 MM-7 | 5–9 | 0.574 | 8, 3742 | 0.800 | Estimable |
| SHARE-W7 ADL-6 | 9 | — | — | — | Not estimable: one interval |
| SHARE-W7 MM-7 | 9 | — | — | — | Not estimable: one interval |

One of six estimable joint tests was significant. In the ELSA ADL-6 waves 4–6 interaction model, the highest-versus-zero wave-specific HRs were 1.93 (95% CI 1.38–2.71) at wave 4, 1.17 (0.72–1.88) at wave 5, and 0.84 (0.45–1.57) at wave 6, suggesting possible concentration in the earliest interval. The six-wave waves 4–9 global test was not significant; because the windows overlap, it is not an independent replication test. The signal remains isolated and multiplicity-unadjusted. Wave-specific estimates were not promoted to primary results.

**Supplementary Table S26C. Standardized absolute-risk sensitivity with age and age squared in both cause-specific models**

| **Analysis** | **Risk, category 0 (95% CI)** | **Risk, highest category (95% CI)** | **RD, percentage points (95% CI)** | **RR (95% CI)** | **RD change from linear-age model** | **RR relative change** |
| --- | --- | --- | --- | --- | --- | --- |
| ELSA ADL-6, 6 years | 19.8% (18.3–21.2) | 26.9% (21.9–32.8) | +7.1 (+1.8 to +13.2) | 1.36 (1.09–1.69) | +0.0 | +0.2% |
| ELSA MM-7, 6 years | 21.6% (19.9–23.3) | 27.9% (22.3–34.9) | +6.3 (+0.3 to +13.5) | 1.29 (1.01–1.64) | −0.3 | −1.0% |
| SHARE-W3 ADL-6, 6 years | 14.4% (11.0–17.9) | 18.9% (17.7–20.4) | +4.5 (+0.8 to +8.5) | 1.31 (1.05–1.75) | 0.0 | −0.1% |
| SHARE-W3 MM-7, 6 years | 28.9% (24.4–34.0) | 36.5% (34.1–39.2) | +7.6 (+1.7 to +12.8) | 1.26 (1.05–1.53) | −0.4 | −1.3% |
| SHARE-W7 ADL-6, 2 years | 4.3% (3.0–6.2) | 7.2% (6.4–8.3) | +2.9 (+0.9 to +4.7) | 1.66 (1.14–2.52) | +0.2 | +4.9% |
| SHARE-W7 MM-7, 2 years | 12.4% (9.9–15.9) | 11.2% (10.0–12.9) | −1.2 (−4.8 to +2.0) | 0.90 (0.69–1.21) | −0.2 | −1.9% |

The largest absolute change in an RD was 0.4 percentage points, and the largest relative change in an RR was 4.9%. Thus, allowing nonlinear baseline age in both the outcome and death models did not materially change the Table-3 interpretation.

**Supplementary Table S26D. ELSA lagged ADL-6 sensitivity after an observed ADL-6-free wave-4 landmark**

| **Follow-up window** | **N** | **Person-intervals** | **Events** | **Competing deaths** | **Highest versus 0 HR (95% CI)** | **p** | **Corresponding original-landmark HR** | **Relative HR change** |
| --- | --- | --- | --- | --- | --- | --- | --- | --- |
| Waves 5–6, mortality-ascertained | 3,598 | 6,646 | 460 | 140 | 1.03 (0.70–1.52) | 0.885 | 1.43 (1.09–1.87), waves 4–6 | −27.9% |
| Waves 5–9, mortality-incomplete after wave 6 | 3,598 | 13,145 | 860 | 140 | 1.27 (0.98–1.64) | 0.070 | 1.47 (1.19–1.82), waves 4–9 | −13.6% |

The lagged analysis required an observed ADL-6-free wave-4 assessment and at least one later observable ADL-6 assessment or known death. It therefore conditions on survival, wave-4 response, remaining outcome-free, and later observability. Its attenuation is consistent with the original association appearing strongest in wave 4 but cannot distinguish temporal attenuation from selection into the later landmark.

**Supplementary Table S26E. SHARE sensitivity to alternative within-country scaling of the baseline weight**

| **Analysis** | **Released country-specific scale, HR (95% CI)** | **Country mean=1, HR (95% CI)** | **Equal country total, HR (95% CI)** | **Largest relative HR change** | **CI exclusion of 1 changed?** |
| --- | --- | --- | --- | --- | --- |
| SHARE-W3 ADL-6 | 1.36 (1.08–1.71) | 1.20 (1.02–1.42) | 1.20 (1.01–1.43) | −11.8% | No |
| SHARE-W3 MM-7 | 1.34 (1.09–1.64) | 1.28 (1.12–1.46) | 1.28 (1.12–1.46) | −4.7% | No |
| SHARE-W7 ADL-6 | 1.60 (1.06–2.43) | 1.54 (1.20–1.98) | 1.63 (1.26–2.11) | −3.7% | No |
| SHARE-W7 MM-7 | 0.93 (0.68–1.27) | 1.14 (0.93–1.40) | 1.22 (0.97–1.54) | +31.3% | No |

Country mean=1 gives each represented country a mean participant-level baseline weight of 1 and leaves its aggregate contribution proportional to analytic sample size. Equal country total gives each represented country the same aggregate baseline-weight mass. These are diagnostics of the pooled common-slope summary, not alternative population targets or country-specific effect models. Four of eight scaled point estimates changed by at least 10%; most notably, the uncertain SHARE-W7 MM-7 estimate changed direction while all CIs continued to include 1.

**Supplementary Table S26F. Sensitivity of SHARE standardized absolute risks to country-level baseline-weight scaling**

| **Inception sample** | **Outcome** | **Horizon** | **Weight scale** | **Risk at score 0, % (95% CI)** | **Risk at score 2, % (95% CI)** | **Risk difference, percentage points (95% CI)** | **Risk ratio (95% CI)** |
| --- | --- | --- | --- | --- | --- | --- | --- |
| SHARE wave-3 inception sample | ADL-6 disability | 6 years | Released scale | 14.4 (11.4–17.9) | 18.9 (17.8–20.3) | 4.5 (0.7–8.0) | 1.32 (1.04–1.69) |
| SHARE wave-3 inception sample | ADL-6 disability | 6 years | Country mean = 1 | 14.5 (12.4–17.1) | 16.9 (16.0–18.1) | 2.5 (−0.5–4.8) | 1.17 (0.97–1.40) |
| SHARE wave-3 inception sample | ADL-6 disability | 6 years | Equal country total | 14.2 (11.9–16.7) | 16.9 (15.9–18.0) | 2.7 (0.0–5.5) | 1.19 (1.00–1.46) |
| SHARE wave-3 inception sample | MM-7 multimorbidity | 6 years | Released scale | 28.6 (24.2–33.6) | 36.6 (34.2–39.0) | 8.0 (2.4–13.1) | 1.28 (1.07–1.52) |
| SHARE wave-3 inception sample | MM-7 multimorbidity | 6 years | Country mean = 1 | 29.7 (26.6–33.1) | 34.9 (33.0–37.0) | 5.3 (1.2–8.7) | 1.18 (1.04–1.32) |
| SHARE wave-3 inception sample | MM-7 multimorbidity | 6 years | Equal country total | 29.6 (26.4–33.0) | 35.5 (33.7–37.4) | 5.9 (2.0–9.8) | 1.20 (1.06–1.37) |
| SHARE wave-7 inception sample | ADL-6 disability | 2 years | Released scale | 4.5 (3.1–6.6) | 7.1 (6.4–8.1) | 2.6 (0.4–4.4) | 1.58 (1.06–2.36) |
| SHARE wave-7 inception sample | ADL-6 disability | 2 years | Country mean = 1 | 4.0 (3.2–5.0) | 6.0 (5.6–6.5) | 2.0 (0.9–3.0) | 1.51 (1.18–1.91) |
| SHARE wave-7 inception sample | ADL-6 disability | 2 years | Equal country total | 3.5 (2.9–4.5) | 5.6 (5.2–6.3) | 2.1 (1.0–3.0) | 1.59 (1.24–1.99) |
| SHARE wave-7 inception sample | MM-7 multimorbidity | 2 years | Released scale | 12.3 (9.6–15.8) | 11.2 (10.0–13.0) | −1.0 (−4.7–2.3) | 0.92 (0.70–1.24) |
| SHARE wave-7 inception sample | MM-7 multimorbidity | 2 years | Country mean = 1 | 11.2 (9.7–13.5) | 12.5 (11.6–13.5) | 1.3 (−1.3–3.3) | 1.11 (0.91–1.33) |
| SHARE wave-7 inception sample | MM-7 multimorbidity | 2 years | Equal country total | 10.9 (9.1–13.6) | 12.9 (12.0–14.2) | 2.0 (−0.9–4.3) | 1.18 (0.94–1.46) |

Values are standardized cumulative incidence estimates with 95% CIs. SHARE score 0 denotes neither of the two cohort-specific childhood disadvantages, and score 2 denotes both material and cultural disadvantage. The released scale retains the country-specific scale of the exposure-wave calibrated SHARE weight. Country mean = 1 preserves within-country relative weights but makes aggregate country mass proportional to analytic sample size. Equal country total preserves within-country relative weights but assigns every represented country equal aggregate baseline-weight mass. For each variant, the same baseline-weight scale was used in the baseline-weight × IPCW cause-specific models and in standardization over the landmark risk set. Models included linear baseline age, sex, country, interval-time-band adjustment, actual interval duration, first observed onset, and death as a competing event.

Released-scale rows reproduce the frozen script-12 reporting output used in Table 3, including its stored point estimates and 95% CIs. Alternative-scale intervals used 1,000 multivariate-*t* draws generated within this diagnostic from the joint survey-linearized outcome/death covariance; IPCW estimation, scaling factors, and the empirical standardization distribution were treated as fixed. This was an unplanned post-review diagnostic. Alternative scales do not identify a preferred population target or estimate country-specific effects. Under alternative scaling, the SHARE-W3 ADL-6 RD attenuated and its interval included or approached 0; the SHARE-W3 MM-7 and SHARE-W7 ADL-6 contrasts remained positive. The SHARE-W7 MM-7 point estimate changed from a negative to a positive RD, while all three intervals remained compatible with no difference. The six-year W3 and two-year W7 risks are not directly comparable.

*Provenance: Tables S26A–B use `01_scripts/17_model_form_sensitivity.R` and `03_results/model_form_age_quadratic_comparison.csv`, `model_form_exposure_wave_global_tests.csv`, `model_form_exposure_wave_high_hr_by_wave.csv`, and `model_form_sensitivity_validation_checks.csv`. Table S26C uses `01_scripts/19_age_quadratic_competing_risk_absolute_risks.R` and `03_results/age_quadratic_competing_risk_primary_comparison.csv`. Table S26D uses `01_scripts/18_elsa_lagged_adl_sensitivity.R` and `03_results/elsa_lagged_adl_sensitivity_high_exposure_summary.csv`. Table S26E uses `01_scripts/20_share_country_weight_scaling_sensitivity.R` and `03_results/share_country_weight_scaling_high_exposure.csv`. Tables S26C–E are governed by `00_protocol/SAP_amendment_05_final_statistical_closure_2026-07-27.md`. Table S26F uses `01_scripts/21_share_country_scaled_competing_risk_absolute_risks.R`, `03_results/share_country_weight_scaling_absolute_supplement_table.csv`, and `share_country_weight_scaling_absolute_validation_checks.csv` under `00_protocol/SAP_amendment_06_SHARE_country_scaled_absolute_risks_2026-07-27.md`.*

### 27. Quality-gate summary

**Supplementary Table S27. Final automated quality gate**

| **Check group** | **Result** |
| --- | --- |
| Derived-data validation | 114/114 |
| SHARE raw-state correction | 11/11 |
| Participant-flow reconciliation | 24/24 |
| Unique combined survey-design × IPCW analyses | 8/8 |
| Finite and ordered combined estimates | 8/8 |
| Independent-administrative-truncation IPCW sensitivity | 12/12 |
| Administrative-censor comparison | 4/4 |
| Finite and ordered competing-risk contrasts | 80/80 |
| Weighted competing-risk validation | 22/22 |
| Weighted competing-risk administrative-truncation validation | 12/12 |
| Fatal SHARE fieldwork and weight audit checks | 19/19 |
| SHARE weight-candidate checks | 4/4 |
| Primary-model covariate completeness | 6/6 |
| Parent-education true-landmark risk-set checks | 14/14 |
| Parent-education raw-state checks | 14/14 |
| Complete and unique side-by-side key-result rows | 48/48 |
| Finite and ordered side-by-side estimates | 48/48 |
| SHARE source-to-landmark selection checks | 10/10 |
| SHARE longitudinal-weight full-chain checks | 11/11 |
| ELSA indicator-specific sensitivity checks | 17/17 |
| Model-form diagnostic checks | 10/10 |
| ELSA lagged ADL-6 sensitivity checks | 14/14 |
| Age-quadratic competing-risk absolute-risk checks | 24/24 |
| SHARE country-scale weight diagnostic checks | 44/44 |
| SHARE country-scaled absolute-risk checks | 31/31 |
| Fatal error markers in analysis logs | None detected |

The original core gate contained 41 gate assertions, all of which passed. The eight targeted closure analyses added 161 gate assertions, all of which also passed. The larger numerators in Table S27 are component-level validations summarized by those gate assertions.

*Provenance: `03_results/final_quality_gate.csv`, `share_source_to_landmark_validation_checks.csv`, `share_longitudinal_weight_full_chain_validation_checks.csv`, `elsa_component_sensitivity_qa.csv`, `model_form_sensitivity_validation_checks.csv`, `elsa_lagged_adl_sensitivity_validation_checks.csv`, `age_quadratic_competing_risk_validation_checks.csv`, `share_country_weight_scaling_validation_checks.csv`, and `share_country_weight_scaling_absolute_validation_checks.csv`.*

## Supplementary Figures

**Supplementary Figure S1. Estimator triangulation for first observed ADL-6 disability and MM-7 multimorbidity.**

Five estimators compare the cohort-specific highest category with zero: available-follow-up unweighted, monotone unweighted, monotone plus IPCW, baseline survey-weighted, and baseline-weight × IPCW. Inference is participant-cluster robust for the first three and uses the declared survey-design approximation for the last two. ELSA waves 4–9 is mortality-incomplete, and the ELSA and SHARE contrasts are not equivalent doses. Values match Supplementary Tables S14A–B.

**Supplementary Figure S2. ELSA indicator-specific associations and composition of the highest exposure category.**

Panel A shows separate and mutually adjusted product-weight cause-specific hazard ratios for the three ELSA indicators in the mortality-ascertained waves 4–6 window. Models used the same outcome-specific risk sets and covariate structure as the main ELSA analysis; the mutually adjusted models included all three indicators simultaneously. Panel B shows the four mutually exclusive indicator combinations among the 439 participants with scores of at least 2. These exploratory analyses clarify exposure content but do not identify a unique causal component. Panel A values are reported in Supplementary Table S25B and Panel B values in Table S25A; standardized risk differences are reported separately in Table S25C and are not plotted.

**Supplementary Figure S3. SHARE selection before the health landmark and sensitivity to the adjacent-wave longitudinal weight.**

Panel A shows the proportion of wave-3 and wave-7 SHARELIFE source respondents who completed the next regular health interview, by the same-interview two-indicator exposure category. Panel B compares the exposure-wave cross-sectional and adjacent exposure-to-landmark longitudinal weight candidates within the otherwise identical baseline-weight × post-landmark IPCW analysis chain. The similar hazard ratios do not remove pre-landmark selection: adjacent-wave longitudinal weights remain conditional on survival and participation at the health landmark and cannot recover respondents who died or did not participate. Panel A values are reported in Supplementary Tables S23A–B and Panel B hazard ratios in Table S24A; the corresponding absolute-risk sensitivity estimates are reported separately in Table S24B.

## Supplementary interpretation

Across primary product-weight analyses using the declared available survey-design information, the highest cohort-specific exposure category was positively associated with first observed ADL-6 onset in ELSA, SHARE-W3, and SHARE-W7. The corresponding primary absolute-risk contrasts were also positive and were insensitive to adding age squared to both cause-specific models, although the SHARE-W3 ADL-6 absolute contrast attenuated under alternative country-weight scaling. The ELSA ADL-6 association nevertheless appeared strongest in the first post-landmark interval: after requiring participants to remain observed and ADL-6-free at wave 4, the lagged waves 5–6 estimate was imprecise and compatible with no association and the waves 5–9 estimate was smaller and imprecise. MM-7 results depended more strongly on the analysis window and inception sample: the association was positive in mortality-ascertained ELSA waves 4–6 and SHARE-W3, attenuated in the mortality-incomplete ELSA waves 4–9 window, and unclear in SHARE-W7 over its single two-year follow-up.

These patterns do not demonstrate why associations differ across cohorts. Differences may arise from exposure content, baseline composition, recall, outcome measurement, follow-up window, survey design, country contribution to pooled weighting, attrition, mortality selection, or other cohort and period differences. The SHARE country-scale diagnostics changed several relative and absolute point estimates. HR uncertainty classifications were unchanged, but the alternative-scale SHARE-W3 ADL-6 RD intervals included or approached 0, and the uncertain SHARE-W7 MM-7 HR and RD point estimates changed direction while remaining compatible with no association. Selective survival and institutional context therefore remain untested hypotheses rather than established mechanisms.

## Abbreviations

ADL, activities of daily living; CI, confidence interval; ELSA, English Longitudinal Study of Ageing; ESS, effective sample size; HR, hazard ratio; IPCW, inverse probability-of-censoring weighting; ISCED, International Standard Classification of Education; MM, multimorbidity; PSU, primary sampling unit; RD, risk difference; RR, risk ratio; SHARE, Survey of Health, Ageing and Retirement in Europe; W, wave.

## Reproducibility and provenance index

The frozen revision analysis is documented in the accompanying analysis-code/SAP archive. All provenance paths below are relative to the archive root. The principal files supporting this supplement are:

- analysis plan: 00_protocol/Revision_SAP_v2_2026-07-23.md;
- SHARE raw-state amendment: 00_protocol/SAP_amendment_02_SHARE_raw_coverscreen_states_2026-07-23.md;
- ELSA indicator-specific amendment: 00_protocol/SAP_amendment_03_ELSA_indicator_specific_exposure_2026-07-25.md;
- SHARE landmark-selection/longitudinal-weight amendment: 00_protocol/SAP_amendment_04_SHARE_landmark_selection_longitudinal_weight_2026-07-25.md;
- final statistical-closure amendment: 00_protocol/SAP_amendment_05_final_statistical_closure_2026-07-27.md;
- SHARE country-scaled absolute-risk amendment: 00_protocol/SAP_amendment_06_SHARE_country_scaled_absolute_risks_2026-07-27.md;
- missingness and parental-education memo: 00_protocol/baseline_missingness_and_share_parent_education_memo_2026-07-23.md;
- weighted competing-risk method: 00_protocol/weighted_competing_risk_absolute_risk_method_2026-07-23.md;
- participant flow: 03_results/sample_flow_detailed.csv and 03_results/no_followup_reasons.csv;
- follow-up status: 03_results/wave_followup_status.csv;
- exposure distributions: 03_results/elsa_exposure_distribution.csv and 03_results/share_exposure_distribution.csv;
- missingness: 03_results/baseline_covariate_mi_decision.csv;
- survey/IPCW design: 03_results/survey_ipcw_combined_weight_design_diagnostics.csv and 03_results/survey_ipcw_combined_response_model_diagnostics.csv;
- estimator comparison: 03_results/key_results_comparison.csv;
- main product-weight estimates: 03_results/survey_ipcw_combined_results.csv;
- absolute risks: 03_results/weighted_competing_risk_absolute_risks.csv and 03_results/weighted_competing_risk_absolute_contrasts.csv;
- SHARE weight sensitivity: 03_results/share_cross_sectional_vs_longitudinal_weight_hr_comparison.csv;
- SHARE exposure-to-landmark selection: 03_results/share_source_to_landmark_overall.csv and 03_results/share_source_to_landmark_by_exposure.csv;
- SHARE longitudinal-weight full chain: 03_results/share_longitudinal_weight_full_chain_summary.csv;
- ELSA indicator-specific sensitivity: 03_results/elsa_component_separate_hazards.csv, elsa_component_mutually_adjusted_hazards.csv, and elsa_component_absolute_contrasts.csv;
- model-form diagnostics: 03_results/model_form_age_quadratic_comparison.csv and model_form_exposure_wave_global_tests.csv;
- ELSA lagged ADL-6 sensitivity: 03_results/elsa_lagged_adl_sensitivity_high_exposure_summary.csv;
- age-quadratic standardized absolute-risk sensitivity: 03_results/age_quadratic_competing_risk_primary_comparison.csv;
- SHARE within-country baseline-weight scaling: 03_results/share_country_weight_scaling_high_exposure.csv;
- SHARE country-scaled absolute risks: 03_results/share_country_weight_scaling_absolute_supplement_table.csv and share_country_weight_scaling_absolute_validation_checks.csv;
- administrative censoring: 03_results/survey_ipcw_combined_share_admin_censor_handling_sensitivity.csv;
- parental education: 03_results/share_parent_education_true_landmark_results.csv;
- final audit: 03_results/final_quality_gate.csv.

The machine-readable CSV files retain unrounded estimates and diagnostics. Values in this document are rounded only for presentation.
